# A Rapid One-Pot Workflow for Sensitive Microscale Phosphoproteomics

**DOI:** 10.1021/acs.jproteome.3c00862

**Published:** 2024-07-22

**Authors:** Gul Muneer, Ciao-Syuan Chen, Tzu-Tsung Lee, Bo-Yu Chen, Yu-Ju Chen

**Affiliations:** † Institute of Chemistry, Academia Sinica, Taipei 11529, Taiwan; ‡ Institute of Biochemical Sciences, National Taiwan University, Taipei 10617, Taiwan; § Chemical Biology and Molecular Biophysics Program, Taiwan International Graduate Program, Academia Sinica, Taipei 11529, Taiwan; ∥ Department of Chemistry, National Taiwan University, Taipei 10617, Taiwan

**Keywords:** Phosphoproteomics, Data-Independent Acquisition, Sample Preparation, Lung Cancer, EGFR-Tyrosine Kinase Inhibitor (TKI)

## Abstract

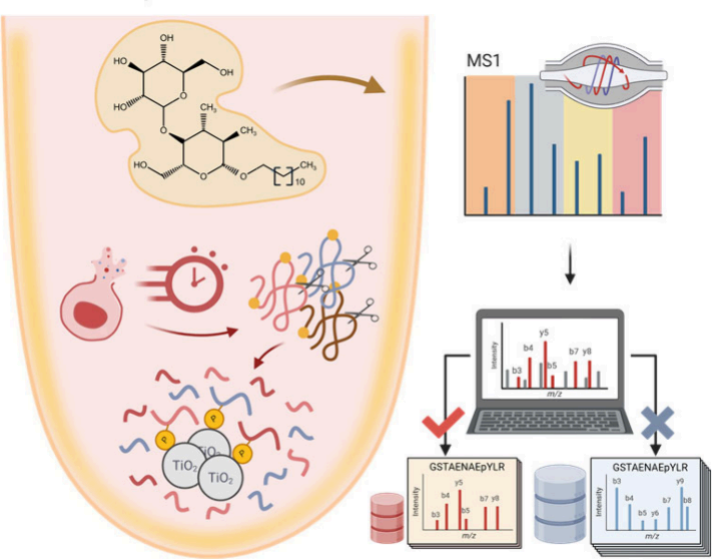

Compared to advancements in single-cell proteomics, phosphoproteomics sensitivity has lagged behind due to low abundance, complex sample preparation, and substantial sample input requirements. We present a simple and rapid one-pot phosphoproteomics workflow (SOP-Phos) integrated with data-independent acquisition mass spectrometry (DIA-MS) for microscale phosphoproteomic analysis. SOP-Phos adapts sodium deoxycholate based one-step lysis, reduction/alkylation, direct trypsinization, and phosphopeptide enrichment by TiO_2_ beads in a single-tube format. By reducing surface adsorptive losses via utilizing *n*-dodecyl β-d-maltoside precoated tubes and shortening the digestion time, SOP-Phos is completed within 3–4 h with a 1.4-fold higher identification coverage. SOP-Phos coupled with DIA demonstrated >90% specificity, enhanced sensitivity, lower missing values (<1%), and improved reproducibility (8%–10% CV). With a sample size-comparable spectral library, SOP-Phos-DIA identified 33,787 ± 670 to 22,070 ± 861 phosphopeptides from 5 to 0.5 μg cell lysate and 30,433 ± 284 to 6,548 ± 21 phosphopeptides from 50,000 to 2,500 cells. Such sensitivity enabled mapping key lung cancer signaling sites, such as EGFR autophosphorylation sites Y1197/Y1172 and drug targets. The feasibility of SOP-Phos-DIA was demonstrated on EGFR-TKI sensitive and resistant cells, revealing the interplay of multipathway Hippo-EGFR-ERBB signaling cascades underlying the mechanistic insight into EGFR-TKI resistance. Overall, SOP-Phos-DIA is an efficient and robust protocol that can be easily adapted in the community for microscale phosphoproteomic analysis.

## Introduction

Protein phosphorylation is a prevalent post-translational modification that creates postgenomic diversity and dynamically regulates various signaling pathways and cellular processes that maintain physiological functions.^1,2^ Aberrant phosphorylation-mediated signaling networks have been closely associated with the initiation and progression of diseases. Mapping global protein phosphorylation events has revealed a system-wide view of cellular signaling networks and provided comprehensive understanding of disease mechanisms.^3^ In the example of cancer, many kinase inhibitors have been approved for cancer targeted treatment through blocking their aberrant phosphorylation signaling pathways, including 71 small-molecule kinase inhibitors (SMKIs) approved by the Food and Drug Administration (FDA) and 16 SMKIs approved by other therapeutic agencies from 2001 to 2021.^4,5^

More recently, advancements in mass spectrometry instrumentation along with substantial improvements in sample preparation have greatly promoted the proteome profiling sensitivity to the single-cell level, facilitating illumination of cellular phenotypes and biological states comprising complex biological systems.^6−8^ Compared to the proteome profiling, however, such small scale phosphoproteome analysis has been challenging due to the demand of sufficient materials that typically requires 100–200-fold more starting amounts. This is primarily due to the low phosphorylation stoichiometry, lower abundance of site-specific phosphopeptides among predominant presence of unmodified peptides (estimated <1% of total abundance), lower mass spectrometry detectivity, and sample loss during the more complex phosphoproteomic workflow compared to proteomics. Efforts to establish a high performance phosphoproteomics protocol for low sample input down to the microscale (μg sample) is imperative to promote its application in challenging samples such as sorted immune cells, rare type blood cells, or biopsy tissues.

To achieve comprehensive phosphoproteome coverage, conventional workflows typically tailored for bulk samples require milligrams to hundreds of micrograms of starting sample amounts.^6−9^ These workflows undergo cell/tissue processing, protein extraction, precipitation, detergent removal prior to protein digestion, fractionation, phosphopeptide enrichment, and multiple sample desalting processes, and are thus prone to sample loss due to multiple sample transfer steps, large processing volumes, and long processing times. A pioneering study by Masuda et al. reported the first microscale phosphoproteomic characterization of 1,011 unique phosphorylation sites from 2 μg proteins (10,000 HeLa cells) by hydroxy acid-modified metal oxide chromatography (HAMMOC) for phosphopeptide enrichment followed by a miniaturized LC column-coupled LC-MS/MS system.^10^ Post et al. reported an automatic workflow by using commercial Fe-IMAC cartridges on a robotic platform that enabled identification of 1,443–4,541 phosphopeptides from 1 μg–10 μg peptides (5 × 10^3^– 5 × 10^4^ cells).^11^ Similarly, Leutert et al. reported a magnetic particle-based automatic workflow on a commercial robotic platform (R2-P2) and achieved a sensitivity of 4,000 distinct phosphopeptides from 25 μg proteins (1.2 × 10^5^ cells).^12^ By eliminating protein precipitation step in a 96-well plate workflow (EasyPhos), Humphrey et al. reported a high-throughput platform with a depth of 20,132 phosphopeptides from 200 μg (1 × 10^6^ cells)EGF-stimulated glioblastoma cell proteins, and ∼4,000 phosphopeptides from a lower input (12.5 μg (6.25 × 10^4^ cells) proteins).^13^ Chen et al. reported a spintip-based approach (Phospho-SISPROT) by integrating three tips for protein digestion, Ti^4+^-IMAC for phosphopeptide enrichment and C18 membrane for peptide desalting, which enabled identification of 600–5,500 phosphopeptides from 1–20 μg (5 × 10^3^– 1 × 10^5^ cells) pervanadate treated HEK 293T cell digests.^17^ More recently, Tsai et al. reported a tandem tip-based approach (C18-IMAC-C18 workflow) by integrating three tips for pre-enrichment cleanup (C18 disk), Fe-IMAC for phosphopeptide enrichment and second C18 disk for post enrichment cleanup which boosted sensitivity to 3,000–9,500 phosphopeptides from 1–10 μg proteins (5 × 10^3^ – 5 × 10^4^ cell).^14^ These works demonstrated significantly improved phosphoproteomic profiling sensitivity through specialized LC setup, robotic handling equipment, and tandem sample processing systems, which may require special instrumentation to be broadly accessible in other laboratories.

The major challenge for microscale phosphoproteomics is the substantial sample loss due to lengthy sample processing workflows and tube surface adsorption during multistep sample transfer, a fundamental issue that has not been fully addressed. Learning from the evolution of single-cell proteomics technologies, streamlining sample processing, and mitigating surface adsorptive losses of proteins and peptides from the mass-limited sample significantly enhance their profiling sensitivity that allows realization of single-cell proteomic profiling.^15,16^ To address this inherent challenge and devise a simple protocol that can be easily adapted by most nonexpert laboratories, in this study, we developed a rapid and simplified one-pot phosphoproteomic workflow (SOP-Phos) coupled with sample size-compatible library-based DIA for highly sensitive microscale phosphoproteomic profiling. First, we evaluated feasibility of three lysis buffers (sodium deoxycholate (SDC), urea and RapiGest) to design one-step lysis, reduction and alkylation, followed by direct trypsinization and phosphopeptide enrichment using TiO_2_ beads in a single-tube format to minimize processing steps. Second, we optimized digestion time from overnight to 2 h, allowing the completion of whole sample processing within 3–4 h. Third, we utilized *n*-dodecyl β-d-maltoside (DDM) to precoat the surfaces of sample processing and collecting tubes which further reduced the sample loss from protein/peptide adsorption on the tube surfaces. SOP-Phos coupled to DIA-MS demonstrated unprecedented sensitivity, enhanced coverage, <1% missing values, and good quantitative reproducibility. Finally, size-comparable spectral library DIA further enhanced 2.6–6.4-fold more phosphopeptides from 5 μg to 0.5 μg cell lysate and 2.0–7.0-fold more phosphopeptides from 50,000 to 2,500 cells, in comparison to direct DIA. We also evaluated the sensitivity for in-depth mapping of phosphorylation sites in signaling pathways and druggable targets. The performance of SOP-Phos-DIA was further demonstrated in the mechanistic study of EGFR-TKI-resistant and sensitive lung cancer cell lines. With the use of commonly used reagents and instrumentation, efficient processing and high profiling sensitivity, this protocol can be easily adapted for daily operation by nonexpert users in the general community.

## Experimental Section

### Materials and Reagents

Phosphate buffer saline (PBS, 10 mM sodium phosphate, 140 mM NaCl, pH 7.4), sodium deoxycholate (SDC), sodium lauroyl sarcosinate (SLS), dithiothreitol (DTT), iodoacetamide (IAM), triethylammonium bicarbonate (TEABC), tris (2-carboxyethyl) phosphine hydrochloride (TCEP), 2-chloroacetamide (CAA), aqueous ammonium hydroxide, phosphatase inhibitor cocktail 2, and phosphatase inhibitor cocktail 3, 2,5-dihydroxybenzoic acid (DHB), trifluoroacetic acid (TFA), and formic acid (FA) were purchased from Sigma-Aldrich (St. Louis, MO, USA). RapiGest SF surfactant was purchased from Waters (MA, USA). Urea was purchased from USB corporation (Cleveland, OH USA). MS grade Lysyl endopeptidase (Lys-C) and trypsin were purchased from FUJIFILM Wako Pure Chemical Corporation (Wako, Osaka, Japan) and Promega (Madison, WI, USA), respectively. Titanium dioxide (TiO_2_) beads were purchased from GL Sciences (Cat No. 5010-75010, Tokyo, Japan). Styrene divinylbenzene (SDB-XC) Empore and C8 membranes were purchased from 3M (St. Paul, MN, USA). The Pierce BCA Protein Assay Kit was purchased from Thermo Fisher Scientific.

### Cell Culture

The NSCLC (PC9) cell line was obtained from the RIKEN BioResource Center (Cat No. RCB4455) and stocked at our institution. The lung cancer cell lines CL68, H1975, and H3255 were obtained from Dr. Pan-Chyr Yang, National Taiwan University Hospital at Taipei, Taiwan. Cells were cultured in RPMI-1640 medium supplemented with (10%, v/v) fetal bovine serum (FBS), sodium bicarbonate (2%, w/v), sodium pyruvate (1 mM), and 1% Antibiotic-Antimycotic solution (Gibco, USA) at 37 °C in a humidified atmosphere of 5% CO_2_ and 95% air.

### Preparation of DDM-Coated Vials

We added 150 μL of 0.01% DDM solution into Eppendorf Protein LoBind tubes (0.5 mL). The tubes were placed on an ELMI Intelli-mixer (RM-2L) and incubated overnight at room temperature (set with mode F8 at 40 rpm). Then, tubes were centrifuged at 1,000*g* at room temperature. The DDM solution was discarded, and the tubes were stored at 4 °C until further use for sample preparation.

### Cell Lysis and Protein Digestion

PC9 cells (10 cm dish, 10^6^) were bathed with ice-cold PBS at least three times. Next, cells were lysed with 0.5 mL of cocktail lysis buffer containing either (1) 1% SDC or (2) 0.3% RapiGest or (3) 8 M urea, and 10 mM TCEP, 40 mM CAA, phosphatase inhibitors (PP2 and PP3) in 100 mM Tris-HCl pH 9.0. Then, lysate (lysed cells) was heated at 95 °C for 5 min and sonicated with 5 cycles of pulse and pause for 5 min at 4 °C, and the supernatant was collected after centrifugation at 16000*g* for 30 min at 4 °C. Protein concentration was measured through BCA assay, and the desired protein amount (0.5 μg - 10 μg) was adjusted in a final volume of <10 μL with 0.1 M Tris-HCl. For overnight digestion, proteins were digested with Lys-C and trypsin at an enzyme to substrate ratio of (Lys-C, 1:100; trypsin, 1:50). For digestion optimization experiments (4-, 2- and 1-h digestion duration), proteins were digested with Lys-C and trypsin at an enzyme to substrate ratio of (Lys-C, 1:20; trypsin, 1:10), and the reaction mixture was incubated with constant shaking (2,000 r.p.m.) at 37 °C for 4, 2, or 1 h. After proteolytic digestion, an equivalent volume of isopropanol (IPA) was introduced (final volume = ∼20 μL) to prevent precipitation of SDC in the subsequent step. The prevention of SDC precipitation eliminated the need for a pre-enrichment desalting step, thereby reducing both sample loss and processing steps.

For control experiments using a conventional protocol, the cells were lysed with 0.5 mL by phase transfer surfactants (PTS) buffer containing 12 mM SDC, 12 mM SLS, 100 mM Tris-HCl (pH 9.0), phosphatase inhibitor cocktail, and protease inhibitor.^17^ Then, lysate (lysed cells) was boiled at 95 °C for 5 min and sonicated with 5 cycles of pulse and pause for 5 min at 4 °C. After centrifugation at 16,000*g* for 30 min at 4 °C, the supernatant was collected, and detergents were removed via methanol-chloroform precipitation method. The extracted proteins were solubilized in 8 M urea, and the protein concentration was measured by BCA assay. The desired amount was subjected to reduction and alkylation using DTT and IAM for 30- and 45 min incubation at 29 °C. Then, proteins were digested with Lys-C and trypsin at an enzyme to substrate ratio of (Lys-C, 1:100; trypsin, 1:50), and the reaction mixture was incubated with constant shaking (2,000 r.p.m.) at 37 °C for overnight (16 h). After enzymatic digestion, the reaction was stopped by adding a final concentration of 0.5% TFA. The peptides were desalted with SDB-XC membrane packed in D200 StageTip. Briefly, the membrane was preactivated with Buffer B (80% ACN, 0.1% TFA) and then conditioned with Buffer A (5% ACN, 0.1% TFA), and peptide samples were passed by centrifuging at 500*g* for 5 min at 25 °C. After washing with Buffer A, the peptides were eluted with Buffer B and collected in the LoBind tube.

For low-cell input experiments, the cell density was measured with an automated cell counter (TC20, BIO-RAD). Then, cells were serially diluted to prepare 50,000, 25,000, 12,500, 5,000, and 2,500 cells in PBS. Before cell lysis, cells were centrifuged at 100*g* for 10 min, and PBS was aspirated to 10 μL. Then, 20 μL of cocktail lysis buffer was added for cell lysis and protein extraction.

### Phosphopeptide Enrichment by TiO_2_ Beads

Phosphopeptide enrichment was performed as follows: After addition of IPA, an equivalent volume of enrichment buffer (3.2 M Lactic acid, 60% ACN, 0.5% TFA) was added (final volume ∼40 μL) and mixed thoroughly. Next, TiO_2_ beads suspended in buffer B at concentration of 75 μg/μL were added into the sample and incubated with shaking (1,500 r.p.m) at 25 °C for 5 min. The titanium-bound phosphopeptides were pelleted down by centrifuging at 1,000*g* for 30 s, and supernatants containing free-peptides were discarded. Then, titanium beads were resuspended in 50 μL wash buffer C (3.2 M lactic acid, 60% ACN, 0.1% TFA) and transferred to a prepacked C8 membrane D200-StageTip. Another round of 50 μL wash buffer C was added into the sample containing tube to collect remaining beads and transferred to a C8 membrane D200-StageTip. After centrifugation at 1,000 r.p.m. for 1 min to pass the solution through the tip, beads were washed 3 times with buffer B to wash out bound lactic acid and free-peptides. After the final wash, phosphopeptides were eluted with 50 μL 0.5% piperidine into a tube precontaining TFA (final concentration, 0.5%) and desalted through a reversed-phase D200-StageTip with SDB-XC as discussed above.

For the control experiment using a conventional protocol, a C8 membrane D200-StageTip was packed with TiO_2_ beads (1.5 mg/60 μL) and equilibrated with 60 μL of buffer B and C. Then an equal volume of buffer C (100 μL) was added into a clean peptide sample (100 μL in buffer B) and passed through the tip by centrifuging at 1,000 r.p.m. for 2 min. After washing with buffer C (1 time) and buffer B (2 times), phosphopeptides were eluted with 100 μL 0.5% piperidine and desalted through a reversed-phase StageTip (SDB-XC packed D200-StageTip). Then, clean peptides were dried with a speed vac evaporator concentrator and stored at −30 °C until analysis. The dried phosphopeptides were reconstituted in a 5 μL MS loading buffer (0.1% formic acid) spiked with indexed retention time (iRT) standard peptides (Biognosys, Schlieren, Switzerland), and 4.5 μL was injected to LC-MS/MS.

### LC-MS/MS Analysis

All the LC-MS/MS analyses were performed on an Orbitrap Fusion Lumos Tribrid mass spectrometer (Thermo Fisher Scientific) coupled with a Thermo Fisher Scientific UltiMate 3000 RSLCnano system via a nanoelectrospray source. The injected phosphopeptides were separated on a reversed phase Waters nanoEase M/Z Peptide CSH C_18_ column of 25 cm length, 75 μm inner diameter, packed with 1.7 μm particles of a 130 Å pore size at 250 nL/min using buffer A (0.1% FA in water) and buffer B (0.1% FA in ACN). The loaded peptides were separated with a gradient of 2%–25% buffer B in 71 min, followed by a 4 min increase to 40% buffer B and to 90% buffer B in 76 min and held for 4 min at 90% buffer B for a column wash. The column was then re-equilibrated at 2% buffer B for 10 min before the next injection. The mass spectrometer was operated in positive mode with an electrospray voltage of 1700 V, the RF lens level was set to 30%, and ion transfer tube was heated at 275 °C for desolvation. For DDA mode, top N multiply charged precursors were automatically isolated and fragmented according to their intensities within the cycle time of 3 s. The intensity threshold was set to 8E3. Full MS was scanned at a resolution of 120,000 with an automatic gain control (AGC) target of 4E5 and a max injection time of 50 ms. The mass range was set to 400–1,250 *m*/*z*, and the isolation width for MS/MS analysis was set to 1.4 *m*/*z* with advanced peak determination. Normalized collision energy (CE) of high-energy collision dissociation (HCD) was set to 30%. MS/MS was scanned in an orbitrap at a resolution of 30,000 with an AGC target of 5E4 and a max injection time of 54 ms. For sample amounts above 10 μg sample (bulk amount), full MS was scanned at a resolution of 60,000 and the MS/MS orbitrap resolution was set to 15,000.

The DIA data were acquired with the following parameters: scan range = 400–1,250 *m*/*z*, MS orbitrap resolution of 120,000 at 200 *m*/*z*, automatic gain control (AGC) target = 4E5, and maximum injection time (IT) = 50 ms. The precursor mass range was set to 500–1,000 *m*/*z*, and 50 windows of 10 Da isolation window were used with an overlap of 1 Da. Subsequently, MS/MS spectra were obtained in the higher-energy collisional dissociation (HCD) with the following parameters: normalized collision energy = 30%, scan range = 110–1,600 *m*/*z*, MS/MS orbitrap resolution = 30,000, AGC target = 5E4, and IT = 54 ms.

### Data Analysis

The DIA raw files were analyzed using Spectronaut v18 software in a library-free workflow (classic direct DIA) as well as in a home-built spectral libraries workflow (library DIA) with default settings unless otherwise stated. For both dirDIA and libDIA, the identification search was performed against the UniProt human proteome database (UniProt Reference Proteome, Taxonomy 9606, Proteome ID UP000005640, 20,387 entries, downloaded 23-09-2021) containing iRT peptide sequences. The search parameters of Spectronaut were set as follows: Trypsin/P as a digestion enzyme, peptide length from 7 to 52, maximum missed cleavages were set to 2, carbamidomethyl on cysteine as static modification, acetyl at protein N-terminus, oxidation at methionine, phosphorylation on S/T/Y as variable modifications, and maximum modifications were set to 5. The false discovery rate (FDR) was set to 0.01 at the precursor, peptide, and protein level. The phosphosite localization algorithm implemented in Spectronaut was activated, and the localization probability cutoff was set to 0 to evaluate localization probability distribution or 0.75 to filter class-1 localized sites. Cross run normalization was set to automatic; interference correction was enabled for both MS1 and MS2, minimum relative fragment intensity was set to 5% (default in Spectronaut 18 is 1%), best intense fragment ions was set to minimum 5 and maximum 15 per spectrum, and the decoy generation method “mutated” based on neural network (NN) predicted fragments was used. Following Spectronaut processing, the peptide and site reports for all searches were exported for further statistics and bioinformatics analysis.

The DDA files were analyzed using MSFragger (v3.8) via FragPipe (v20.0).^18^ The search parameters of FragPipe were set as follows: Full tryptic digestion with maximum missed cleavages was set to 2. Carbamidomethyl (C) was set as a fixed modification, and oxidation at methionine (M), acetylation (protein N-term), and Phospho (STY) was set as variable modifications for maximum of five variable modifications. The final reports were filtered at a 1% peptide spectrum match (PSM)/peptide and 1% protein level for further analysis. Match between runs (MBR) was turned off to search peptides generation from individual runs.

### Spectral Library Construction

The project-specific sample small-size spectral libraries were constructed by using the Pulsar search engine under Spectronaut v 18 (Sagan) software (Biognosys, Zurich, Switzerland). For sensitivity evaluation, a small library was constructed from 10 μg using PC9 cells, and the data were acquired from DDA (*n* = 3) and DIA (*n* = 3). For differential profiling of TKI-sensitive and resistant cells experiment, additional data acquired from 1 μg using CL68, H1975, PC9, and H3255 cells (DIA, *n* = 4) were combined with 10 μg library to generate a sample-specific library. Both DDA and DIA data files were imported into Spectronaut and processed using default settings unless specified otherwise. Following settings were used for library generation in Spectronaut: The FDR cutoff was set to 1% at the PSM, peptide, and protein level, activating phosphosites localization probability. The precursors with phosphorylation modifications were finally retained in the library. Carbamidomethylation was set as fixed modification and methionine oxidation, acetylation (at protein N terminus), and phosphorylation of serine, threonine, and tyrosine (STY) were set as a variable modification with a maximum of 5 modifications. The enzyme parameter was set to Trypsin/P for up to two missed cleavages. The peptide length was set to 7–52 in the search space. The best most intense fragment ions (minimum = 5 and maximum = 15) with a relative intensity of 5% (default in Spectronaut 18 is 1%) per spectrum were included, whereas the fragment ions with less than three amino acid residues were not considered.

### Data Interpretation and Statistical Analysis

A minimum of three technical or biological replicates were used to generate the data in this study. The Pearson’s correlation coefficient was calculated on log_10_-transformed intensities. All the statistical analysis was performed by Perseus software (2.0.11). For EGFR-TKI-sensitive and resistant lung cancer cells experiment, phosphosites had a localization probability ≥0.75, and their abundances were log_2_-transformed for further analysis. Statistically significant changes in phosphosite abundances were calculated by an FDR controlled two-sample *t* test (permutation-based FDR < 0.01 and S0 = 0). The signaling pathways enrichment analysis was performed using DAVID (2021, http://david.ncifcrf.gov). For kinase-substrate enrichment analysis, kinase activity was inferred from differentially regulated phosphosites as previously described.^19^ Significantly different phosphosites from both resistant cell pairs were used to query the PhosphositePlus and NetworKIN database. The substrate cutoff was set to 5, and the FDR was set to 0.05 to generate *z*-scores for kinase-substrate enrichment. All the data are shown as mean ± SD from triplicate analyses. The bar height in the bar plot shows an average number of three replicates. The gray circles over bar plots indicate the numbers from individual replicates. The box in each box plot encapsulates the interquartile range (IQR), with the bottom and top edges representing first (Q1) and third Q3, respectively. The median is marked by a horizontal line within the box.

## Results and Discussion

### Design of a Rapid and Simplified Phosphoproteomic Workflow in One-Pot

**Figure 1:**
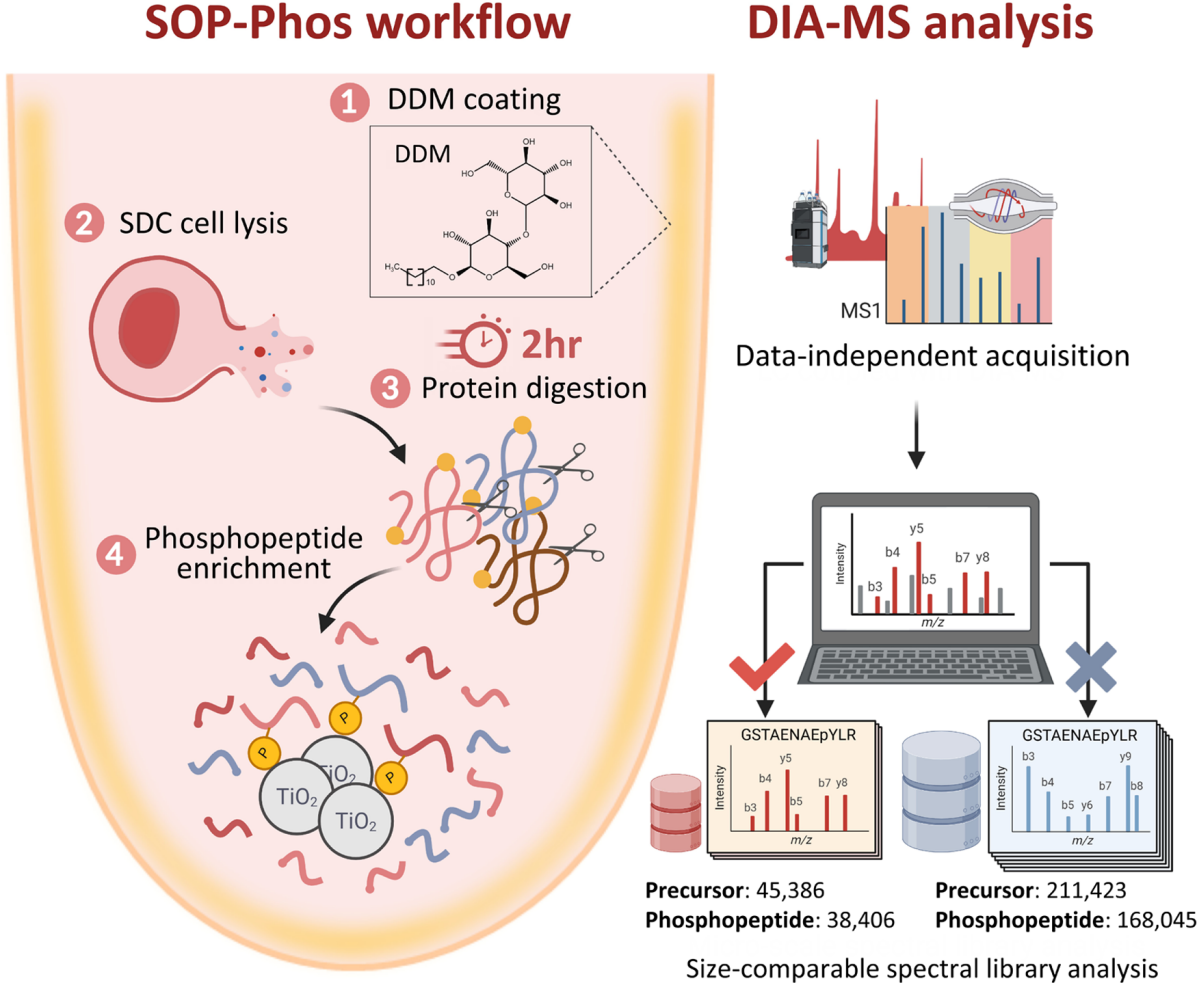
Schematics of simple and rapid one-pot phosphoproteomics workflow (SOP-Phos) integrated with DIA-MS for microscale phosphoproteomic analysis. (A, B) SOP-Phos protocol capitalizes DDM to precoat the surfaces of sample processing and collecting tubes, employs SDC-based one-pot processing, reduces the volume by 3-fold (50 μL) compared to conventional workflow, shortens processing time to 3–4 h, and is coupled with sample size-comparable library DIA to enable sensitive microscale phosphoproteome profiling.

To minimize sample loss, we adapted two strategies. First, prior to the experiment, we exploited DDM to preblock active surfaces on Protein LoBind tubes throughout sample processing and collection to prevent adsorptive losses of proteins and peptides from the tube surface (*Step 1*, Figure 1A). DDM, a nonionic detergent that adsorbs on hydrophobic surfaces, has been previously employed by spiking a small amount into collection vials at the end of the sample preparation.^20^ Second, the SOP-Phos (simple and rapid one-pot phosphoproteomics) protocol was designed as a substantially simplified workflow of only three steps: cell lysis, protein digestion, and phosphopeptide enrichment. The conventional workflows employ powerful chaotropic agents, such as phase transfer surfactant (PTS),^17^ guanidine hydrochloride (GdmCl) buffers,^6^ or sodium laurate^7^ to lyse cells and extract and solubilize proteins. In these protocols, multiple desalting steps are essential to remove these reagents because they interfere with tryptic digestion, reversed-phase separation, and phosphopeptide enrichment. However, the resulting complex multistep processing significantly increases the sample loss for size-limited samples.

Our protocol streamlined sample processing by combining sodium deoxycholate (SDC) detergent and reducing/alkylating buffer to concurrently perform cell lysis, protein reduction, and alkylation in a single step (*Step 2,*Figure 1A). We further shortened the conventional overnight enzymatic digestion by increasing the enzyme-to-protein ratio (from 1:50 to 1:10) to reduce the digestion time to 1–2 h, allowing completion of the whole protocol within 3–4 h (*Step 3,*Figure 1A). Following enzyme digestion, titanium dioxide beads are directly added into tryptic peptides for phosphopeptides enrichment (*Step 4*), thereby circumventing the need for sample cleanup prior to enrichment. The entire workflow is completed within 3–4 h with minimal sample processing steps.

We recently reported a sample-size comparable spectral library-based DIA approach to boost coverage of low abundance proteins.^21^ In contrast to the large-scale comprehensive spectra library, scaling the library size with a comparable sample amount improved mapping of low-abundance peptides in low-input samples. Compared to proteome characterization, where a protein has multiple peptides, the identification of site-specific phosphorylation relies only on a limited number of mass spectra from a single sequence. Thus, its success is critically determined by the spectra similarity between the library and experimental DIA-based fragmentation spectra. To boost the profiling coverage for the microscale (microgram level, μg) phosphoproteomics, we established small high-quality spectral libraries with different levels of sample input to enable in-depth library-based DIA profiling of the microscale samples (Figure 1B).

### One-Pot Phosphoproteomic Protocol Boosts Microscale Profiling Sensitivity

**Figure 2:**
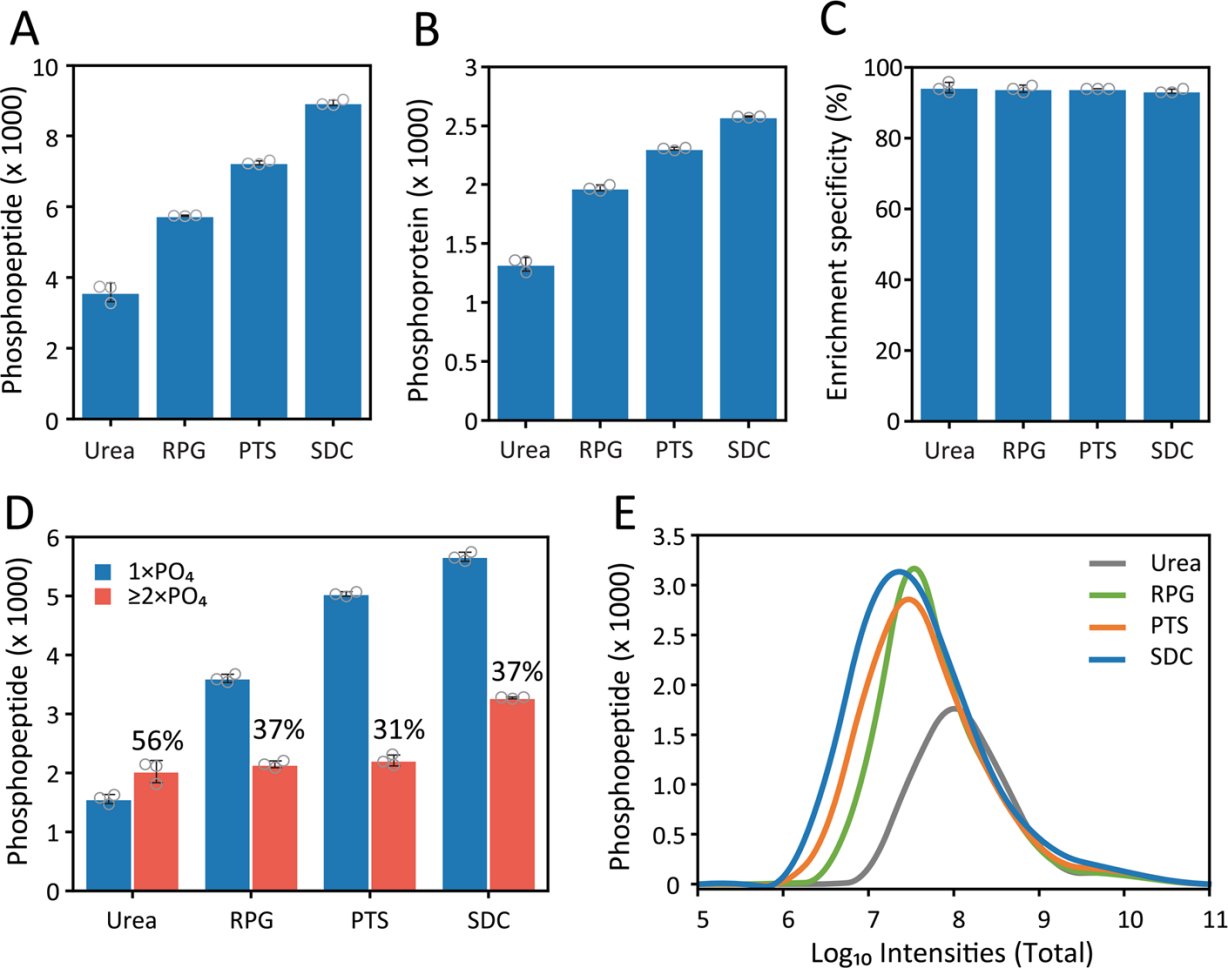
Optimization of the sample preparation protocol for microscale phosphoproteome analysis. (A, B) Number of phosphopeptides and phosphoproteins identified by four sample preparation protocols (urea, RapiGest, PTS, and SDC) for phosphoproteome analysis. (C) Ratio of phosphopeptides to total peptides identified by each workflow. (D) Number of singly and multiply phosphorylated peptides (containing more than one phosphate groups) identified in different workflows. (E) Distribution of log_10_-transformed intensities of total phosphopeptides. All the data are shown as the mean ± SD from technical triplicate analyses, and gray dots indicate the identification from individual replicates.

To develop a rapid, one-pot workflow with minimal steps, we evaluated three detergents, including urea, RapiGest (designated RPG in the Figure 2), and sodium deoxycholate (SDC), as the cell lysis buffers to leverage their compatibility with the subsequent tryptic digestion and direct phosphopeptide enrichment by TiO_2_. We combined detergent (Urea, RapiGest, or SDC) with reducing (Tris(2-carboxyethyl) phosphine, TCEP) and alkylating agent (chloroacetamide, CAA) to concurrently perform cell lysis, protein reduction, and alkylation in a single step. This facilitates bypassing the protein-precipitation and detergent removal steps prior to enzymatic digestion, as well as the cleanup step before phosphopeptide enrichment. The protocol was also compared with the conventional protocol, which utilizes phase transfer surfactant (PTS) lysis buffer and necessitates detergent removal through protein precipitation and peptide desalting before phosphopeptide enrichment. Although DDM surfactant has been employed for one-pot single-cell proteomics, we excluded DDM as a lysis buffer due to the need for a large volume and high concentration of DDM to lyse a relatively larger number of cells (10^6^ cells), which may damage the LC-column, as reported previously.^22^

In this study, PC9 cells (∼5 × 10^6^ cells) were used to evaluate protein extraction efficiency by different workflows. We observed that SDC-based lysis yielded the highest protein extraction, outperforming all other buffers (1.6, 1.5, and 1.2-fold increase compared to RapiGest, urea, and PTS) (Figure S1A). Consistently, the SDC-based protocol enabled the highest phosphopeptide (8,946 ± 78) coverage (Figure 2A and Table S1), yielding an average of 60%, 36%, and 19% more phosphopeptides than urea (3,585 ± 261), RapiGest (5,753 ± 14), and PTS (7,251 ± 55) buffers, respectively. The superiority of SDC-based protocol was also observed in achieving the highest coverage of phosphoproteins (Figure 2B). Moreover, the overall improved identification also led to an elevated number of class-1 sites (4,986 ± 8) compared to other protocols (1748 ± 148, 2798 ± 210, 3686 ± 59) in urea, RapiGest, and PTS-based workflows, respectively (Figure S1B). Notably, all other workflows showed high enrichment specificity (>90%), revealing that none of these buffers compromised the enrichment specificity of the TiO_2_-based enrichment workflow. In summary, the result demonstrates robustness and stability of the approach despite the presence of diverse reagent backgrounds (Figure 2C). Interestingly, diverse chemical backgrounds influenced the enrichment of singly and multiply phosphorylated peptides. SDC-based protocol identified more multiply phosphorylated peptides (3,276 ± 15) than urea (2,025 ± 185), RapiGest (2,148 ± 60), and PTS (2,215 ± 91) based protocols. Notably, this was not at the expense of singly phosphorylated peptides, unlike urea-based protocol, which detected lower monophosphorylated peptides compared to multiply phosphorylated peptides (Figure 2D).

To evaluate phosphopeptide recovery, we also quantified the intensities of all phosphopeptides from the 4 methods. Based on the distribution of abundance index, the comparison shows that the SDC protocol has the broadest abundance range of intensity distribution, covering the lowest abundant peptides compared to other methods (Figure 2D), although their commonly identified phosphopeptides exhibit similar median intensities (Figure S1C). We further explored peptide characteristics to evaluate whether phosphopeptides from different protocols exhibit specific physicochemical properties. The results show a similar distribution of peptide hydrophobicity (GRAVY scores) as well as a similar average peptide length in the range of 16–18 amino acids identified by all protocols (Figure S1D,E). These results demonstrate the improved recovery of lower abundant phosphopeptide signal intensity from our simplified one-pot single-step protocol compared to conventional multistep sample preparation methods.

After establishing the streamlined sample processing protocol, we aimed to shorten the overall workflow by shortening the regular overnight digestion step. Although rapid digestion approach has been widely applied in microscale proteomics,^23^ the recommendation of a large input sample requirement (>200 μg) has deterred its exploration in small-scale phosphoproteomic sample preparation. We tested the feasibility of reducing digestion time for microscale samples (<10 μg protein) by increasing the enzyme (trypsin)-to-protein ratio from 1:50 (overnight) to 1:10 and reducing digestion time to 4, 2, and 1 h. A shorter digestion time generated similar phosphopeptide identification results compared to overnight digestion, while both 1-h and 2-h digestion generated slightly higher numbers of unique phosphosites and class-1 sites among all methods (Figure S2A,B and Table S2). Additionally, such short digestion times exhibited adequate digestion efficiency, with a comparable fraction of phosphopeptides with 0 missed cleavages to that of overnight (Figure S2C). Further comparison with recently published phosphoproteomic studies^11,13,14,24,25^ revealed that rapid digestion (∼2 h) in the SOP-Phos workflow showed a slightly improved percentage (67%) of peptides without miscleavages in comparison to traditional overnight digestion (54%–63%) (Figure S2D). Taken together, these results demonstrate the feasibility of the one-pot-based rapid phosphoproteomics workflow for microscale samples within hours.

### DDM Vial Coating Minimize Peptide/Protein Adsorptive Losses

Surface adsorptive losses during the sample preparation process are likely the major issue to affect the sensitivity of small-scale phosphoproteomic analysis. Previously, bovine serum albumin (BSA) and ionic surfactants (e.g., sodium dodecyl sulfate) were employed to minimize surface adsorption for microscale proteomics.^15,22^ However, addition or surface coating with BSA or ionic surfactants is not suitable for phosphoproteomic sample preparation due to interference of their derived peptides or agents against phosphopeptide enrichment by titanium beads. Recently, spiking a nonionic detergent (DDM) into the peptide collection vial was reported to reduce surface adsorptive losses.^20^ We hypothesize that coating DDM may reduce sample losses in every processing tube. In the conventional multistep protocol, however, “spiking” DDM at different stages of sample preparation increases the risk of contamination derived from the remaining concentrated DDM. On the other hand, “pre-coating” DDM and washing away excess amounts is compatible with our single tube format workflow.

To evaluate feasibility of precoating, we coated the surfaces of sample preparation and collection vials (Protein LoBind Tubes, 0.5 mL, Eppendorf) with 150 μL 0.01% DDM solution overnight at room temperature, and the solution was discarded to remove excess DDM. We assessed the effect of DDM coating at two different stages: (1) using coated vials only after phosphopeptide enrichment steps (partial coating), and (2) using coated vials throughout the sample preparation (full coating). The comparison also allowed us to test whether DDM coating will interfere with titanium oxide-based phosphopeptide enrichment. To verify the effectiveness of the DDM coating process, we checked the extracted ion chromatogram and mass spectrum of DDM in uncoated, partially coated, and fully coated experimental conditions. A trace amount of DDM was observed by a mass peak of 1021.61 *m/z,* representing the DDM dimer in both partially and fully coated conditions, confirming the effectiveness of the DDM coating approach (Figure S3A–C).

**Figure 3:**
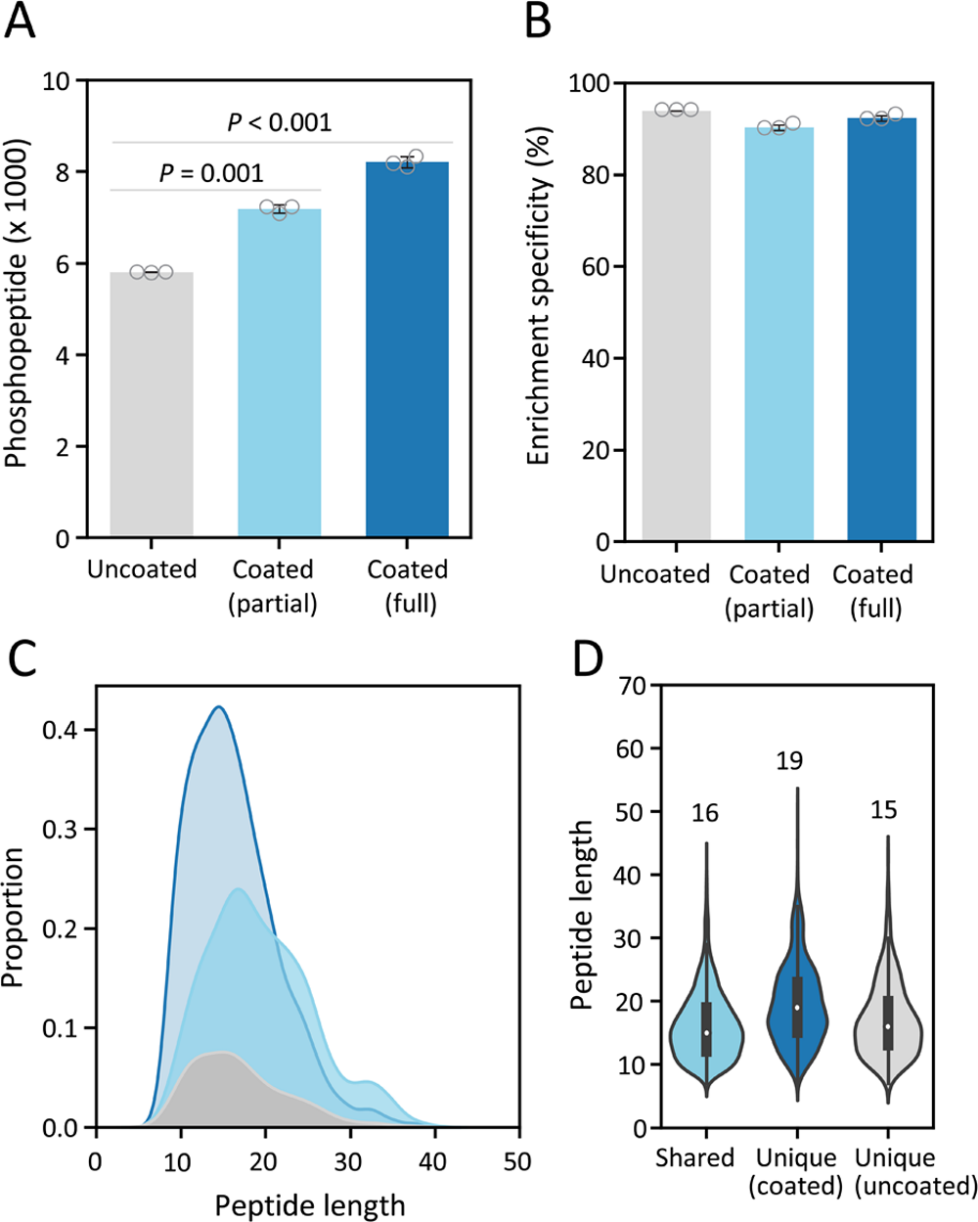
Effect of DDM coating on peptide recovery from low input samples. (A) Comparison of phosphopeptides identified with (partially or fully) and without coating of DDM during phosphoproteomic sample preparation. (B) Ratio of phosphopeptides to total peptides identified by each approach. (C) Distribution of peptide length for phosphopeptides, and (D) those uniquely and commonly identified by both workflows. All the data are shown as mean ± SD from technical triplicate analyses, and gray dots indicate the identification from individual replicates.

By DDM coating, the number of identified phosphopeptides from 2.5 μg (12,500 PC9 cells equivalents) increased by 1.2-fold and 1.4-fold in partially and fully DDM-coated vessels, respectively, compared to uncoated vessels (Figure 3A and Table S3). It is noted that the DDM coating did not interfere with the phosphopeptide enrichment by TiO_2_ beads, achieving similar and high enrichment specificity (90–94%) across all conditions (Figure 3B). The fully DDM-coated approach also covered nearly all (95%) peptides found by uncoated tubes (Figure S4A). Consistently, the phosphopeptides intensities showed a slightly increased median intensity when using DDM-coated tubes (Figure S4B). Multiply phosphopeptides are relatively low in abundance and longer in length compared to the predominantly monophosphopeptides. The results showed that fully DDM-coated tubes recovered slightly more doubly phosphorylated (23.3%) and multiply (2.1%) phosphorylated peptides (Figure S4C), which are likely prone to adsorption on plasticware surfaces, thereby contributing to sample loss and reduced sensitivity. Moreover, the length of the phosphopeptides uniquely identified in DDM-coated tubes is much longer, with an average of 19 amino acids compared to the average of 15 amino acids in those uniquely observed in noncoated tubes (Figure 3C,D), although the distribution of the gravy score showed no significant difference in peptide hydrophobicity between samples prepared in DDM-coated and uncoated tubes (Figure S4D). In summary, DDM coating in our designed single tube-protocol effectively recovered significantly more phosphopeptides, especially the longer and multiple phosphopeptides that are prone to adsorption onto the tube surface.

### Library-Based DIA Enhances Coverage and Reproducibility for Microscale Sample

DIA-MS has rapidly evolved as a promising alternative for achieving reproducible sampling and improved coverage in large-scale phosphoproteomics.^7,26^ To evaluate if DIA, including classic direct DIA (dirDIA) and library-based DIA (libDIA) approaches, also enhances performance of microscale phosphoproteomics, 10 μg and 5 μg cell lysates were used to compare the phosphoproteomic coverage, confidently localized class-1 sites, data completeness, and reproducibility. By the DDA method, an average of 10,994 ± 58 and 9,905 ± 184 phosphopeptides were identified, whereas the direct DIA approach using Spectronaut software enabled higher identification coverage of 13,942 ± 2 and 12,896 ± 3 phosphopeptides from 10 and 5 μg cell lysate, respectively (Figure S5A and Table S4). In particular, the dirDIA method enhanced approximately 2-fold more unique phosphosites, including 1.6-fold confidently localized class-1 (probability ≥0.75) sites, compared to DDA (Figure S5B). In the DDA data set, a large proportion of missing values between runs present a bottleneck for reproducible label-free quantification, particularly in low-input samples.^27^ The comparison also revealed dramatically enhanced reproducibility of SOP-Phos workflow by DIA. The triplicate analysis results showed low overlapping of identified phosphopeptides by DDA (64%–65%), while nearly all (99%–99%%) identified phosphopeptides were reproducibly detected by DIA. Consistently, the comparison of run-to-run variabilities revealed almost no missing values in dirDIA (<1%) compared to DDA (<38%) (Figure S5C). Although DIA enhanced the detection of lower abundance peptides, the phosphopeptides quantified by dirDIA also showed a slightly lower coefficient of variation (8%–10% median CV) (Figure S5D). Taken together, these results demonstrate the advantages of DIA in allowing highly reproducible identification and quantification for microscale sample inputs, which are critical for trace sample analysis.

**Figure 4:**
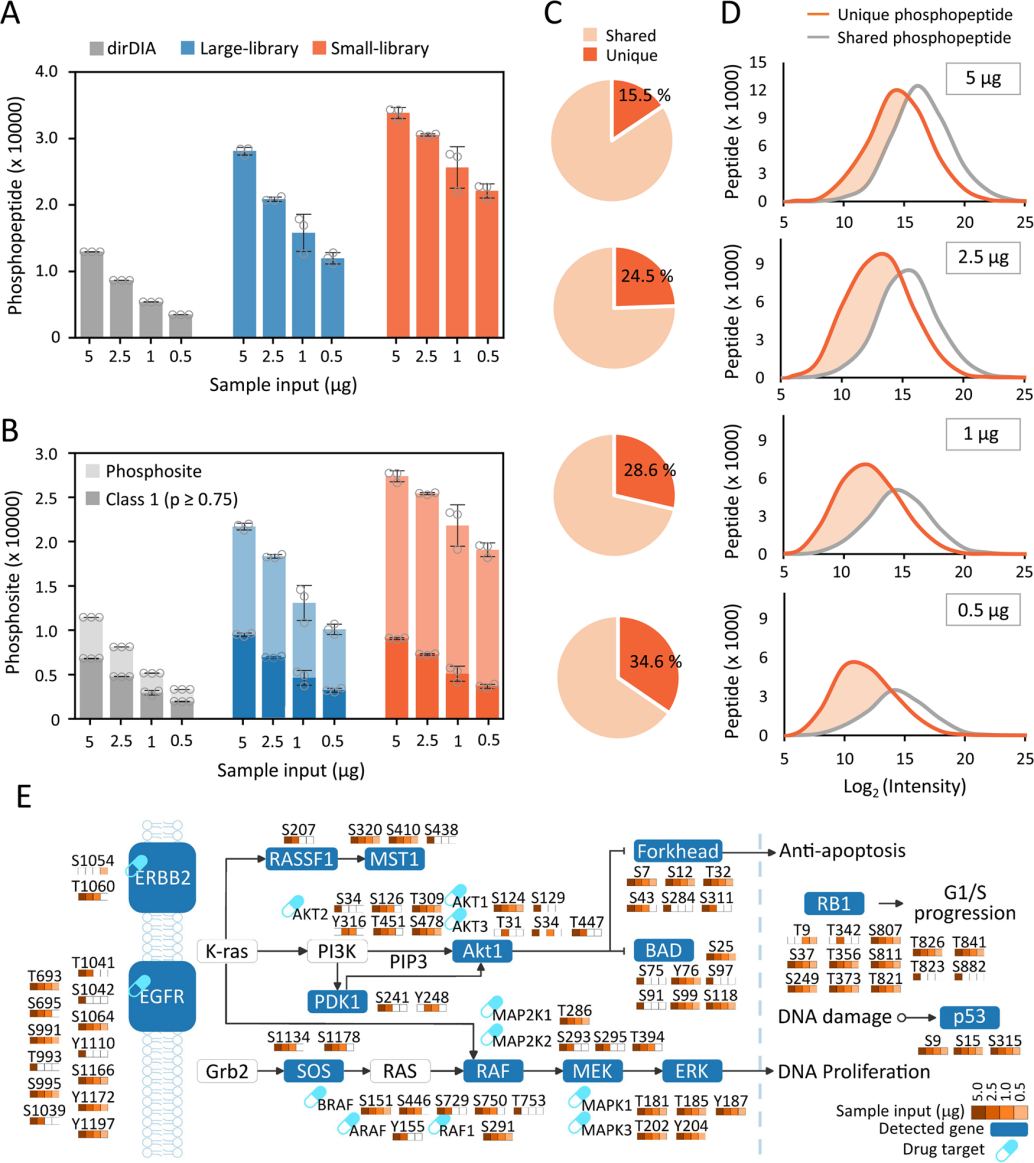
Evaluation of DIA data analysis with spectral libraries generated from different sample sizes. (A, B) Summary of the number of phosphopeptides and phosphosites identified by dirDIA, large library, and small library. (C) Percentage of low-abundance phosphopeptides uniquely identified in small libDIA and shared phosphopeptides identified in small, largelibDIA, and dirDIA. (D) Distribution of phosphopeptide intensities of shared and unique phosphopeptides . (E) Mapping coverage of phosphosites and phosphoproteins in the NSCLC signaling pathway across 0.5–5 μg cell lysate. All the data are shown as mean ± SD from technical triplicate analyses, and gray dots indicate the identification from individual replicates.

Encouraged by the improved performance in direct DIA-based phosphoproteomics coverage, we next evaluated the sensitivity at a lower sample input level. Compared to bulk samples, low-input samples generate significantly lower peptide numbers and abundances, which further change the ion precursor intensity and influence DIA-MS/MS fragmentation patterns during LC-DIA MS data acquisition. Thus, the success of deconvolution and spectral matching in the libDIA approach critically relies on the similarity of fragmentation patterns between the sample and spectral library. We previously highlighted that peptide spectra library established with comparable sample input demonstrated optimal identification coverage.^21^ To further enhance the phosphoproteomics coverage in our SOP-Phos workflow, we evaluated the performance of sample-size comparable spectra library. By dirDIA, an average of 12,896 ± 2.6, 8,602 ± 4.2, 5,369 ± 21.4, and 3,436 ± 0.6 phosphopeptides were identified from 5 μg, 2.5 μg, 1 μg, and 0.5 μg cell lysate, respectively (Figure 4A and Table S5). To further enhance library-based DIA profiling coverage, we established a small sized spectral library and compared its performance with a large sized spectral library. Using 3 DDA and 3 DIA datasets from size-comparable (10 μg) cell lysate, a small library was constructed with a depth of 38,406 phosphopeptides. The large library is based on a previously established, relatively comprehensive lung cancer resource spectral library with lung cancer cell lines and tissue from NSCLC patients, covering a depth of 159,524 phosphopeptides.^7^

In comparison to dirDIA, the comprehensive lung cancer spectral library enhanced 1.9–3.0-fold more phosphopeptides by library-based DIA analysis. The small library had a much more profound effect, particularly for submicrogram samples, significantly enhancing identifications by 2.6, 3.5, 4.8, and 6.4-fold for 5 μg (33,787 ± 670 phosphopeptides), 2.5 μg (30,482 ± 140 phosphopeptides), 1 μg (25,580 ± 2553 phosphopeptides), and 0.5 μg (22,070 ± 861.1 phosphopeptides) cell lysates, respectively (Figure 4A and Table S6). Similarly, the small library achieved 1.2, 1.5, 1.6, and 1.9-fold more phosphosites in comparison to large-scale spectral library DIA (Figure 4B and Table S6). The small libDIA approach covered nearly all (90%–96%) unique phosphopeptides found by dirDIA (Figure S6). The percentage of newly identified low-abundance phosphopeptides by small libDIA was 15.5%, 24.5%, 28.6%, and 34.6% for 5 μg, 2.5 μg, 1 μg, and 0.5 μg cell lysate, respectively (Figure 4C). Interestingly, the peptide abundance distribution showed larger portion of low-abundant peptides that were specifically enriched when using a sample size-comparable library for mapping low-input samples (Figure 4D). These results demonstrate that the appropriate size of the spectral library in proportion to the sample input significantly improves coverage and detection of low-abundance phosphopeptides, particularly for low-input samples.

To further ensure the confidence of gained identifications in library-based DIA, we further examined the FDR and localization probability of identified phosphopeptides from both dirDIA and libDIA using a small library (refer to small libDIA). The Spectronaut software enforces 1% FDR cutoff by default for reliable identification and reports confidence score (calculated by a built-in localization tool in Spectronaut) for localized phosphosites. We observed that FDR (*q*-value) curves appear to converge at approximately 1% FDR for both dirDIA and small libDIA datasets, across low (0.5 μg) and high (5 μg) sample inputs. Although the FDR threshold of small libDIA was higher than that of dirDIA, it still remained under 0.01 for the library-based matches, suggesting the reliability of these hits (Figure S7A–F). However, we observed that phosphopeptides with a relatively higher FDR (passed 1% FDR threshold) in libDIA tend to be assigned with lower localization probability scores (Figure S8A–D). We further examined (1) mass accuracy tolerance and (2) mass error of the matched peptides precursors. In both dirDIA and libDIA, the average mass tolerance for data extraction and scoring varied between 1.08 and 2.72 PPM. This is reflected in the mass error of precursors, which is still within the accepted standard of 3 PPM mass accuracy (Figure S9A–C). These results show that there is no significant difference in the mass tolerance between the dirDIA and libDIA methods. In comparison to proteomics, the identification of phosphopeptides involves two levels: identification of peptide sequence and localizing the phosphorylation site. To further assess the quality of phosphosite localization in dirDIA and small libDIA, we analyzed low (0.5 μg) and high (5 μg) sample inputs using proline (P) phosphorylation as variable modification in which any detected proline phosphorylation is false positive identification.^28^ Both dirDIA and libDIA revealed a significant number of phosphorylation sites confidently localized on serine, threonine, and tyrosine in comparison to proline, demonstrating that library-based DIA can reliably identify and localize phosphorylation sites (Figures S10 and S11). Additionally, the confidence in phosphopeptide identification from library-based matches is evident in their MS/MS spectra for low-abundance proteins such as EGFR (Y1197), YAP1 (T110), and PGRC1 (Y180), which are uniquely identified in small libDIA (Figure S12). Overall, these results suggest the reliability of library-based DIA to improve the phosphoproteomic profiling of low input samples.

With the established sensitivity, we evaluated the identification depth of identified class-1 phosphosites and phosphoproteins to map the coverage in the lung cancer-relevant nonsmall cell lung cancer (NSCLC) pathway across 0.5–5 μg cell input. A total of 79 class-1 phosphosites from 14 phosphoproteins were covered from combined results (Figure 4E). Among these sites, exciting results were achieved with the detection ofY1197 and Y1172 sites on EGFR, the two most important autophosphorylation sites indicating EGFR activation, mutations or amplification, identified in as low as 0.5 μg cell lysate( (2,500 cells equivalents). Notably, important regulatory sites involved in various functions, such as S124 on AKT, activation status markers T202, Y204 onMAPK, activation site S241 on PDK1, and S1178 on SOS1 that disrupts the SOS1-GRB2 interaction, and S151 on RAF to induce its dimerization, were detected across 0.5–5 μg amount. The coverage also includes detection of six FDA-approved drug targets (EGFR, ERBB, BRAF, MAPK1/3, AKT, MAP2K1/3) at different input levels, highlighting the sensitivity of our approach in illuminating the phosphoproteomic profiles from low starting amounts.

In the advancement of phosphoproteomic approaches, most efforts to reduce sample amounts have typically resulted in decreased identification coverage. In the state-of-art single-cell proteomics, the proteome coverage is far lower than the result from the bulk samples, despite the critical importance of achieving deep coverage for deciphering functional networks. To the best of our knowledge, this study achieved superior identification depth and coverage compared to recently published label-free approaches, which reported an identification range of around 600–9,180 phosphopeptides per μg. For-example, Chen et al. developed Phospho-SISPROT, which achieved identification of 600 phosphopeptides from 1 μg lysate. This identifications was further enhanced to 1,443 phosphopeptides by an optimized workflow using commercial cartridges on a Bravo AssayMAP Platform.^11^ Next, Tsai et al. developed a tandem tip-based C18-IMAC-C18 method, which further boosted the coverage to 9,180–18,100 phosphopeptides from 1–10 μg cell lysate by using a library-based approach.^14^ Compared to the above-mentioned studies, this study reported one of the most sensitive phosphoproteome coverages, with the identification of 33,787 ± 670 to 22,070 ± 861 phosphopeptides from 5 μg to 0.5 μg cell lysate. At 1 μg cell input, the coverage of 5,369 ± 21.4 phosphopeptides by direct DIA and 4.8-fold further enhancement to 25,580 ± 2553 phosphopeptides by a small spectra library highlighted the combined strength of loss-less SOP-Phos protocol and sample size-comparable library. Additionally, good analytical merits, including confidently localized sites, good reproducibility, and low missing values, were systematically benchmarked for low-input samples. The unparalleled coverage offered by the SOP-Phos method also enabled the detection of important regulatory sites, such as the relatively low abundance tyrosine phosphorylation sites on EGFR (Y1197/Y1172) and activation/regulatory sites of MAPK, PDK1, AKT and druggable targets (EGFR, ERBB, BRAF, MAPK1/3, AKT, MAP2K1/3), which were previously reported using much higher sample amounts at the phosphorylation level.^7,8^

### Feasibility of Low-Cell Input by SOP-Phos-DIA

**Figure 5:**
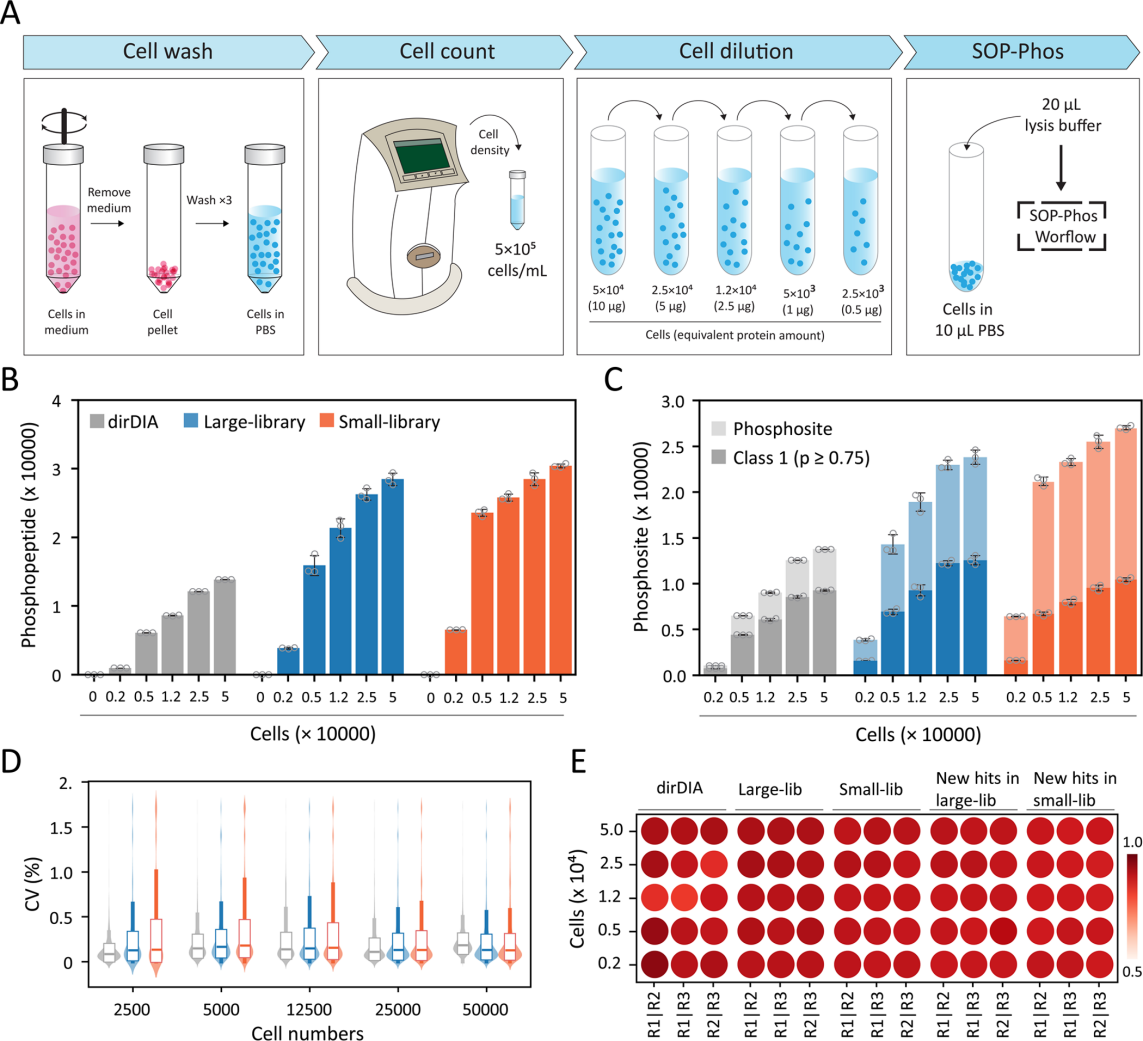
Phosphoproteomic analysis of low-cell input by SOP-Phos-DIA. (A) Overview of PC9 cells preparation and dilution from 50,000 cells to 2,500 cells for SOP-Phos processing. Summary of number of phosphopeptides, (B) and phosphosites (C) including class-1 sites from 0, 2,500, 5,000, 12,500, 25,000, and 50,000 cells identified by dirDIA, large libDIA, and small libDIA. (D) Distribution of coefficient of variation (CV%) for quantified phosphopeptides by dirDIA and library-based DIA. (E) Pearson’s correlation of quantified phosphopeptides in dirDIA, library-based DIA, and uniquely quantified either in the large libDIA or small libDIA. All the data are shown as the mean ± SD from biological triplicate analyses, and gray dots indicate the identification from individual replicates.

Phosphoproteomic profiling of low-cell input samples (i.e., starting from a small number of cells) is a challenging task due to the complex, multistep workflow that causes huge sample loss leading to low identifications. We attempted to assess the capabilities of SOP-Phos workflow for phosphoproteomic profiling using low-cell inputs, ranging from 2,500 cells to 50,000 cells. Following cell wash, the cell density was calculated with an automated cell counter, and cells were serially diluted to prepare 50,000, 25,000, 12,500, 5,000, and 2,500 cells (Figure 5A). Using dirDIA, an average of 974 ± 0, 6,110 ± 4, 8,629 ± 57, 12,127 ± 15, and 13,863 ± 16 phosphopeptides were detected from 2,500 to 50,000 cells with specificity ranging between 85–94%. For the blank sample (0 cells), an average of 12 ± 0 phosphopeptides were identified with dirDIA (Figure 5B and Table S7). Next, we used the same library generated earlier (annotated as large libDIA and small libDIA) to assess phosphopeptide coverage from these low-cell number samples. The small libDIA outperformed both large libDIA and dirDIA in terms of identification. Compared to dirDIA, small libDIA significantly enhanced 2, 2, 3, 4, and 7.0-fold identifications for 50,000, 25,000, 12,500, 5,000 and 2,500 cells, respectively. No phosphopeptides were identified from the blank sample using both libraries under the same FDR, suggesting reliable control of Spectronaut’s library-based DIA approach over the false discovery rate. Both small and large libDIA approaches covered nearly all (93%–95%) unique phosphopeptides found by dirDIA (Figure S13). Compared to dirDIA, the large libDIA enhanced 1.7, 1.8, 2.1, 2.2, and 3.6-fold phosphosites, while the small libDIA has superior sensitivity to cover 2.0, 2.0, 2.6, 3.3 and 6.0-fold phosphosites. Interestingly, the large libDIA mapped slightly higher class-1 phosphosites for 50,000 to 5,000 cells and comparable coverage for the set from 2,500 cells mapped by the small libDIA (Figure 5C and Table S8). This could be attributed to the complete fragmentation pattern and phosphorylated site containing fragments with sufficient abundance in the large libDIA in comparison to the small libDIA. With the small libDIA, the 25,000, 12,500, 5,000, and 2,500 cell samples identified 16%, 15%, 8%, and 70% less phosphopeptides than low input lysate 5 μg–0.5 μg samples.

To evaluate the reproducibility of the SOP-Phos-DIA workflow for the above low-cell input samples, we plotted the distribution of CV and Pearson correlation coefficients for each pairwise comparison between biological replicates. The median CV between replicates were 9–19% from dirDIA, 13–17% from the large libDIA, and 13–18% from the small libDIA in all cell loadings (Figure 5D). Furthermore, we calculated the Pearson correlation of phosphopeptide intensities quantified by dirDIA, large libDIA, small libDIA, and uniquely quantified in both libraries. The results revealed that nearly all Pearson correlation coefficients are ≥0.9 across the analyses, suggesting high reproducibility and consistent quantification (Figure 5E). In summary, SOP-Phos-DIA enables reproducible phosphoproteomic profiling of low-cell input samples.

### Phosphoproteomic Landscape of EGFR-TKI-Sensitive and Resistant Lung Cancer Cells

Epidermal growth factor receptor tyrosine kinase inhibitors (EGFR TKIs) have been the first-line therapy (first and second generation TKIs; gefitinib, erlotinib, and afatinib, third generation TKI, osimertinib) in the treatment of nonsmall cell lung cancer.^29−32^ Though they have proven clinical benefit to prolong survival, most patients eventually develop resistance against EGFR-TKIs, which presents one of the most urgent unsolved burdens. To demonstrate the applications for microscale samples, we applied this method to explore the EGFR-TKI resistant mechanism by quantitative phosphoproteomic profiling of two pairs of TKI-sensitive and TKI-resistant NSCLC cell lines using a 0.5 μg (2,500 cells) starting amount. The gefitinib-sensitive cell lines, PC9 and H3255, harbor the two major EGFR activating mutations, Del19 and L858R mutation, respectively. The gefitinib-resistant cell lines, CL68 and H1975, carry additional acquired resistant mutations, Del19/T790M or L858R/T790M mutations, respectively.

**Figure 6:**
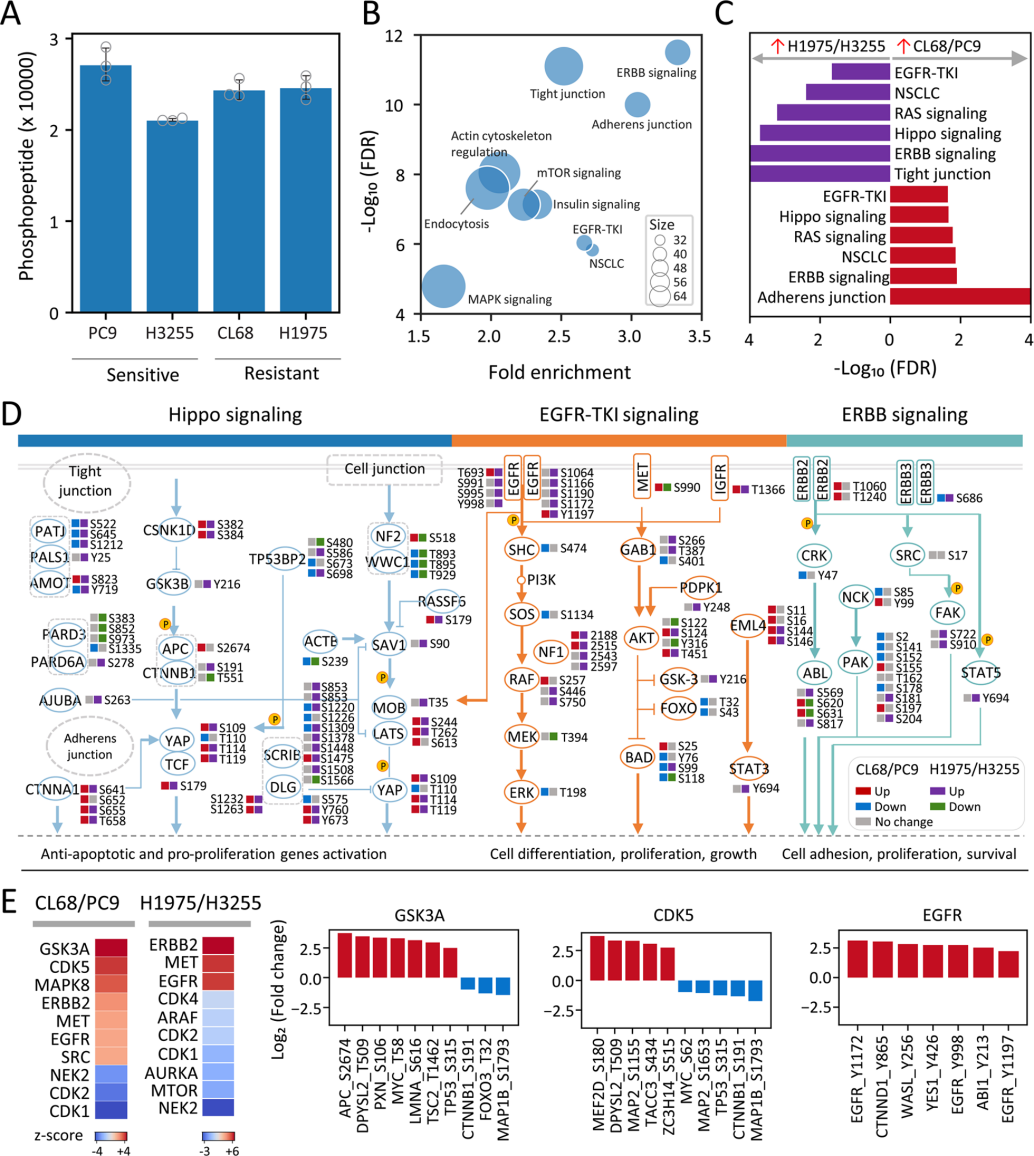
Phosphoproteomic landscape of EGFR-TKI-sensitive and resistant lung cancer cells (A) Summary of phosphopeptides identified in EGFR-TKI-sensitive (PC9 and H3255) and EGFR-TKI-resistant (CL68 and H1975) lung cancer cells. (B) Pathway enrichment analysis of phosphoproteins using the Kyoto Encyclopedia of Genes and Genomes (KEGG) database. (C) KEGG signaling pathways enriched (*p* < 0.05) from upregulated phosphosites/phosphoproteins in EGFR-TKI-resistant CL68 and H1975 lung cancer cells. (D) Phosphoproteomic landscape of Hippo, EGFR-TKI and ERBB signaling pathways, illustrating differentially expressed phosphosites (two-sampled *t*-test, FDR < 0.05). (E) Kinase enrichment using the significantly different phosphosites between resistant and sensitive groups and substrates of kinases with predicted activation (*p* < 0.05). All the data are shown as the mean ± SD from biological triplicate analyses, and gray dots indicate the identification from individual replicates.

With the size-comparable library (10 μg, 40,726 phosphopeptides), an average of 27,194 ± 1,813, 21,131 ± 149, 24,426 ± 1,063, and 24,680 ± 1,298 phosphopeptides were identified from PC9, H3255, CL68, and H1975, respectively (Figure 6A and Table S9). Quantitative comparison of the class-1 phosphosites from the two pairs of CL68/PC9 and H1975/H3255 resulted in 2,743 and 3,142 differentially regulated phosphosites, respectively (two-sample *t*-test, FDR < 0.01, S0 = 0). Among differentially expressed phosphosites, 1,418 and 1,777 phosphosites were upregulated in resistant cells compared to that in sensitive cells. Pathway analysis of all phosphoproteins against the KEGG database enriched the top-ranking pathways, including the NSCLC pathway, EGFR-TKI signaling, ERBB signaling, MAPK signaling, and mTOR signaling, which are all associated with NSCLC signaling (Figure 6B and Table S10). Additionally, adherens junctions, actin cytoskeleton, tight junctions, insulin signaling, and endocytosis were also enriched, which are involved in remodeling and metastasis in cancer. By comparing the pairs of CL68/PC9 and H1975/H3255 cell lines, the pathway analysis of upregulated phosphosites in both resistant cells (CL68, H1975) revealed enriched pathways (*p* < 0.05) related to resistance in lung cancer, including the commonly up-regulated EGFR-TKI resistance pathway, Hippo signaling, and ERBB signaling (Figure 6C and Table S10). Previous studies have reported that Hippo signaling and signaling via the ERBB2 pathway contributed to tumor development and EGFR-TKI resistance.^33,34^ SOP-Phos-DIA provides high coverage to reveal the deep site-specific phosphoproteomic landscape and alterations in phosphosites within these pathways (Figure 6D). In total, 114 phosphosites exhibited differential expression spanning nearly all downstream proteins in all three pathways. As expected, elevated phosphorylation sites in the most well-known EGFR-TKI pathway were observed in both resistant cells, including most well-known autophosphorylation sites (Y1197 and Y1172) upon EGFR activating mutation,^7,35^ along with downstream phosphorylation of the PI3K/AKT signaling cascade due to the acquired T790M mutation in resistant cells. Furthermore, the upregulated phosphorylation at threonine 693 by p38, which leads to the EGFR trafficking for its internalization, has been reported to account for the reduced EGFR-TKI efficacy.^36^

Apart from EGFR, ERBB2 mutation or amplification leads to dimerization with EGFR or ERBB3, which activates downstream PI3K/AKT signaling, has been reported to be involved in gefitinib resistance.^37^ Our results revealed several upregulated phosphorylation events in downstream signaling cascades of the ERBB pathway, including SRC-Y694 mediated activation of STAT5 and phosphorylation of FAK at S910, which regulate cell adhesion, migration, and survival.^38,39^ Furthermore, we found high coverage and alterations in the Hippo signaling pathway. YAP is a core member of the Hippo signaling pathway to regulate cell proliferation, survival, and differentiation.^34^ Interestingly, resistant cells displayed increased phosphorylation of YAP-S109, S119, MOB-T35, and RASSF1A-S179 compared to the sensitive cells, which collectively play roles in YAP activation.^40−42^ Ando et al. reported that EGFR activation by mutations or amplification induces YAP activation by promoting MOB phosphorylation.^43^ YAP activation further induces expression of epidermal growth factor receptors including EGFR, ERBB, and production of their ligands, which in turn activates YAP and leads to EGFR-TKI resistance.^44^ Even with submicrogram cell input, our results show good coverage to reveal the system view of such a complex interplay of TKI-resistance mechanism in both resistant cells. These detailed phosphorylation-events strongly suggest the critical roles of YAP-induced resistance to EGFR TKIs, thus representing a promising target to restore sensitivity to targeted therapies.

To explore the upstream kinases responsible for TKI resistance, kinase-substrate analysis of differentially expressed phosphosites from the two pairs of resistant cells was performed. Kinase enrichment analysis identified GSK3A, CDK5, ERBB2, MET, and EGFR as the top-ranking activated kinases in both resistant cells (Figure 6E and Table S10). For example, motif enrichment (Fisher’s exact test, FDR < 0.05) identified 56 upregulated motifs for the most significantly enriched kinase GSK3A (Figure 6E and Table S10). Targeting GSK-3 has been reported as a promising target to overcome the resistance of NSCLC.^45^ Similarly, CDK5 was identified as the top-ranking kinase with 43 upregulated motifs, which aligned with its reported broad role in promoting the tumorigenic pathways in the development and progression of a variety of cancers including lung cancer, and as a valid target for anticancer therapies.^46^ These identified kinase targets and their overactivated substrate phosphorylation may offer an opportunity to design next-line agents to overcome the EGFR-TKI resistance.

## Conclusions

In this study, we developed a simple and rapid one-pot phosphoproteomic sample preparation workflow coupled to data-independent acquisition mass spectrometry (SOP-Phos-DIA) for low-input sample analysis. SOP-Phos-DIA demonstrated excellent profiling performance, including one of the highest coverages of 22,070 ± 861 to 33,787 ± 670 phosphopeptides from as little as 0.5 μg to 5 μg cell lysate, high reproducibility (8–10% CV), low missing value (<1%), and deep pathway coverage. Furthermore, application of SOP-Phos-DIA to low-cell input samples enabled identification of 30,433 ± 284–6,548 ± 21 phosphopeptides from 50,000 to 2,500 cells samples. These results are likely achieved due to the sample lossless SOP-Phos protocol from the DDM-coated LoBind tube, SDC-based one-pot processing, 3-fold reduced volume (50 μL) than the conventional workflow, shortened 3–4 h processing time, and enhanced phosphopeptide identification by sample-size comparable library DIA.

Our findings highlight the superiority of the SDC-based one-pot strategy over other detergents, including urea, RapiGest, and PTS-based sample preparation workflows, in terms of phosphopeptide coverage and recovery of low-abundance phosphopeptides. These improvements come from efficient extraction of proteins and reduced sample processing steps in a one-pot based buffer, thereby mitigating sample losses which is unavoidable in a conventional protein precipitation-based workflow. Traditional proteolytic digestion suffers from autolysis, low efficiency, and long incubation causing sample losses particularly for low-input samples. Optimizing digestion to as short as 2 h was sufficient to effectively digest proteome, thereby enabling the entire workflow to be executed in a few hours. SOP-Phos combined with DIA demonstrated unprecedented sensitivity, coverage, and quantitative reproducibility, which are critical for studying biology when downscaling the sample amount. Using a size-comparable library, our results demonstrated mapping to NSCLC and many druggable targets from as little as 2,500 cells equivalent input. Furthermore, SOP-Phos-DIA demonstrated robust coverage to reveal a complex interplay of Hippo signaling, EGFR-TKI signaling, and ERBB signaling, which are likely involved in the TKI-resistance mechanism in lung cancer cells. The activation of EGFR, ERBB, and MET in both resistant cells (CL68 and H1975) offers a unique opportunity to innovate drug design to target dual or multiple kinases. In summary, the SOP-Phos-DIA workflow can be easily implemented in a proteomics laboratory for routine sample preparation for microscale phosphoproteomics research. This workflow could be integrated into miniaturized devices which may pave the way for studying signal transduction at nanoscale down to single-cell phosphoproteome level.

## Data Availability

All mass spectrometry raw data sets, spectral libraries, and Spectronaut quantification outputs in this study were deposited in jPOST^47^ and ProteomeXchange. The accession numbers are JPST002415 for JPOST and PXD047646 for ProteomeXchange, and the data can be accessed through https://repository.jpostdb.org/preview/1018227641657308e515bec.

## Supporting Information Available

Figure S1. Comparison of total protein yields, class-1 sites, intensities, and peptide properties by different workflows. Figure S2. Optimization of tryptic digestion time for low input amount. Figure S3. Extracted ion chromatogram and mass spectrum of DDM. Figure S4. Overlap, intensities, phosphopeptide types, and hydrophobicity of peptides detected in uncoated and coated tubes. Figure S5. Comparison of identification and quantitation performance by DIA and DDA methods. Figure S6. Overlapped phosphopeptides identified using dirDIA, large-library, and small-library. Figure S7. Comparison of distribution of *q*-values (FDR) and localization probabilities between dirDIA and lib-based DIA. Figure S8. Distribution of FDR based on localization probability score from 0.5 μg and 5 μg sample input by dirDIA and libDIA. Figure S9. The MS1 mass accuracy tolerance and distribution of mass errors of 0.5 μg sample input from dirDIA, large libDIA, and small libDIA. Figure S10 and Figure S11. Summary of FDR and localization probabilities by target and decoy search using dirDIA and libDIA. Figure S12. Summary of monoisotopic distribution, fragment ion spectra, and XIC of phosphopeptides that are uniquely detected from small libDIA. Figure S13. Overlapped phosphopeptides identified using dirDIA, large-library, and small-library (PDF)
Table S1. Summary of identification results from different protocols (XLSX)
Table S2. Summary of identification results from conventional, 4 h, 2 h, and 1 h (XLSX)
Table S3. Summary of identification results DDM coating on peptide recovery (XLSX)
Table S4. Summary of identification results from low input amount by DIA and DDA (XLSX)
Table S5. Summary of identification results from direct DIA (XLSX)
Table S6. Summary of identification results from library DIA (XLSX)
Table S7. Summary of identification results from direct DIA (XLSX)
Table S8. Summary of identification results from library DIA (XLSX)
Table S9. Summary of identification results from PC9, CL68, H1975, and H3255 cells (XLSX)
Table S10. Summary of KEGG pathway analysis of phosphoproteins from 1000 to 10 cells (XLSX)

## References

[ref1] Needham E. J.; Parker B. L.; Burykin T.; James D. E.; Humphrey S. J. Illuminating the dark phosphoproteome. Sci. Signal. 2019, 12, eaau8645 10.1126/scisignal.aau8645. 30670635

[ref2] Johnson J. L.; Yaron T. M.; Huntsman E. M.; Kerelsky A.; Song J.; Regev A.; Lin T.-Y.; Liberatore K.; Cizin D. M.; Cohen B. M.; et al. An atlas of substrate specificities for the human serine/threonine kinome. Nature 2023, 613, 759–766. 10.1038/s41586-022-05575-3. 36631611 PMC9876800

[ref3] Ochoa D.; Jarnuczak A. F.; Viéitez C.; Gehre M.; Soucheray M.; Mateus A.; Kleefeldt A. A.; Hill A.; Garcia-Alonso L.; Stein F.; et al. The functional landscape of the human phosphoproteome. Nat. Biotechnol. 2020, 38, 365–373. 10.1038/s41587-019-0344-3. 31819260 PMC7100915

[ref4] Attwood M. M.; Fabbro D.; Sokolov A. V.; Knapp S.; Schiöth H. B. Trends in kinase drug discovery: targets, indications and inhibitor design. Nat. Rev. Drug Discovery 2021, 20, 839–861. 10.1038/s41573-021-00252-y. 34354255

[ref5] Min H.-Y.; Lee H.-Y. Molecular targeted therapy for anticancer treatment. Exp. Mol. Med. 2022, 54, 1670–1694. 10.1038/s12276-022-00864-3. 36224343 PMC9636149

[ref6] Bekker-Jensen D. B.; Bernhardt O. M.; Hogrebe A.; Martinez-Val A.; Verbeke L.; Gandhi T.; Kelstrup C. D.; Reiter L.; Olsen J. V. Rapid and site-specific deep phosphoproteome profiling by data-independent acquisition without the need for spectral libraries. Nat. Commun. 2020, 11, 787 10.1038/s41467-020-14609-1. 32034161 PMC7005859

[ref7] Kitata R. B.; Choong W.-K.; Tsai C.-F.; Lin P.-Y.; Chen B.-S.; Chang Y.-C.; Nesvizhskii A. I.; Sung T.-Y.; Chen Y.-J. A data-independent acquisition-based global phosphoproteomics system enables deep profiling. Nat. Commun. 2021, 12, 2539 10.1038/s41467-021-22759-z. 33953186 PMC8099862

[ref8] Sharma K.; D’Souza R. C. J.; Tyanova S.; Schaab C.; Wiśniewski J. R.; Cox J.; Mann M. Ultradeep human phosphoproteome reveals a distinct regulatory nature of Tyr and Ser/Thr-based signaling. Cell Rep. 2014, 8, 1583–1594. 10.1016/j.celrep.2014.07.036. 25159151

[ref9] Humphrey S. J.; Azimifar S. B.; Mann M. High-throughput phosphoproteomics reveals in vivo insulin signaling dynamics. Nat. Biotechnol. 2015, 33, 990–995. 10.1038/nbt.3327. 26280412

[ref10] Masuda T.; Sugiyama N.; Tomita M.; Ishihama Y. Microscale phosphoproteome analysis of 10,000 cells from human cancer cell lines. Anal. Chem. 2011, 83, 7698–7703. 10.1021/ac201093g. 21888424

[ref11] Post H.; Penning R.; Fitzpatrick M. A.; Garrigues L. B.; Wu W.; MacGillavry H. D.; Hoogenraad C. C.; Heck A. J. R.; Altelaar A. F. M. Robust, sensitive, and automated phosphopeptide enrichment optimized for low sample amounts applied to primary hippocampal neurons. J. Proteome Res. 2017, 16, 728–737. 10.1021/acs.jproteome.6b00753. 28107008

[ref12] Leutert M.; Rodríguez-Mias R. A.; Fukuda N. K.; Villén J. R2-P2 rapid-robotic phosphoproteomics enables multidimensional cell signaling studies. Mol. Syst. Biol. 2019, 15, e9021 10.15252/msb.20199021. 31885202 PMC6920700

[ref13] Humphrey S. J.; Karayel O.; James D. E.; Mann M. High-throughput and high-sensitivity phosphoproteomics with the EasyPhos platform. Nat. Protoc. 2018, 13, 1897–1916. 10.1038/s41596-018-0014-9. 30190555

[ref14] Tsai C.-F.; Wang Y.-T.; Hsu C.-C.; Kitata R. B.; Chu R. K.; Velickovic M.; Zhao R.; Williams S. M.; Chrisler W. B.; Jorgensen M. L.; et al. A streamlined tandem tip-based workflow for sensitive nanoscale phosphoproteomics. Commun. Biol. 2023, 6, 70 10.1038/s42003-022-04400-x. 36653408 PMC9849344

[ref15] Gebreyesus S. T.; Siyal A. A.; Kitata R. B.; Chen E. S.-W.; Enkhbayar B.; Angata T.; Lin K.-I.; Chen Y.-J.; Tu H.-L. Streamlined single-cell proteomics by an integrated microfluidic chip and data-independent acquisition mass spectrometry. Nat. Commun. 2022, 13, 37 10.1038/s41467-021-27778-4. 35013269 PMC8748772

[ref16] Zhu Y.; Piehowski P. D.; Zhao R.; Chen J.; Shen Y.; Moore R. J.; Shukla A. K.; Petyuk V. A.; Campbell-Thompson M.; Mathews C. E.; et al. Nanodroplet processing platform for deep and quantitative proteome profiling of 10–100 mammalian cells. Nat. Commun. 2018, 9, 882 10.1038/s41467-018-03367-w. 29491378 PMC5830451

[ref17] Masuda T.; Saito N.; Tomita M.; Ishihama Y. Unbiased quantitation of Escherichia coli membrane proteome using phase transfer surfactants. Mol. Cell. Proteomics 2009, 8, 2770–2777. 10.1074/mcp.M900240-MCP200. 19767571 PMC2816013

[ref18] Yu F.; Teo G. C.; Kong A. T.; Fröhlich K.; Li G. X.; Demichev V.; Nesvizhskii A. I. Analysis of DIA proteomics data using MSFragger-DIA and FragPipe computational platform. Nat. Commun. 2023, 14, 4154 10.1038/s41467-023-39869-5. 37438352 PMC10338508

[ref19] Casado P.; Rodriguez-Prados J.-C.; Cosulich S. C.; Guichard S.; Vanhaesebroeck B.; Joel S.; Cutillas P. R. Kinase-substrate enrichment analysis provides insights into the heterogeneity of signaling pathway activation in leukemia cells. Sci. Signal. 2013, 6, rs6 10.1126/scisignal.2003573. 23532336

[ref20] Dou M.; Tsai C.-F.; Piehowski P. D.; Wang Y.; Fillmore T. L.; Zhao R.; Moore R. J.; Zhang P.; Qian W.-J.; Smith R. D.; et al. Automated Nanoflow Two-Dimensional Reversed-Phase Liquid Chromatography System Enables In-Depth Proteome and Phosphoproteome Profiling of Nanoscale Samples. Anal. Chem. 2019, 91, 9707–9715. 10.1021/acs.analchem.9b01248. 31241912 PMC6741344

[ref21] Siyal A. A.; Chen E. S.-W.; Chan H.-J.; Kitata R. B.; Yang J.-C.; Tu H.-L.; Chen Y.-J. Sample Size-Comparable Spectral Library Enhances Data-Independent Acquisition-Based Proteome Coverage of Low-Input Cells. Anal. Chem. 2021, 93, 17003–17011. 10.1021/acs.analchem.1c03477. 34904835

[ref22] Tsai C.-F.; Zhang P.; Scholten D.; Martin K.; Wang Y.-T.; Zhao R.; Chrisler W. B.; Patel D. B.; Dou M.; Jia Y.; et al. Surfactant-assisted one-pot sample preparation for label-free single-cell proteomics. Commun. Biol. 2021, 4, 265 10.1038/s42003-021-01797-9. 33649493 PMC7921383

[ref23] Ctortecka C.; Hartlmayr D.; Seth A.; Mendjan S.; Tourniaire G.; Udeshi N. D.; Carr S. A.; Mechtler K. An automated nanowell-array workflow for quantitative multiplexed single-cell proteomics sample preparation at high sensitivity. Mol. Cell. Proteomics 2023, 22, 100665 10.1016/j.mcpro.2023.100665. 37839701 PMC10684380

[ref24] Chen W.; Chen L.; Tian R. An integrated strategy for highly sensitive phosphoproteome analysis from low micrograms of protein samples. Analyst 2018, 143, 3693–3701. 10.1039/C8AN00792F. 29978859

[ref25] Murillo J. R.; Kuras M.; Rezeli M.; Milliotis T.; Betancourt L.; Marko-Varga G. Automated phosphopeptide enrichment from minute quantities of frozen malignant melanoma tissue. PLoS One 2018, 13, e0208562 10.1371/journal.pone.0208562. 30532160 PMC6287822

[ref26] Martinez-Val A.; Fort K.; Koenig C.; Van der Hoeven L.; Franciosa G.; Moehring T.; Ishihama Y.; Chen Y.-j.; Makarov A.; Xuan Y.; Olsen J. V. Hybrid-DIA: intelligent data acquisition integrates targeted and discovery proteomics to analyze phospho-signaling in single spheroids. Nat. Commun. 2023, 14, 3599 10.1038/s41467-023-39347-y. 37328457 PMC10276052

[ref27] Lazar C.; Gatto L.; Ferro M.; Bruley C.; Burger T. Accounting for the Multiple Natures of Missing Values in Label-Free Quantitative Proteomics Data Sets to Compare Imputation Strategies. J. Proteome Res. 2016, 15, 1116–1125. 10.1021/acs.jproteome.5b00981. 26906401

[ref28] Leijten N. M.; Heck A. J. R.; Lemeer S. Histidine phosphorylation in human cells; a needle or phantom in the haystack?. Nat. Methods 2022, 19, 827–828. 10.1038/s41592-022-01524-0. 35726056

[ref29] Yang C.-Y.; Yang J. C.-H.; Yang P.-C. Precision Management of Advanced Non-Small Cell Lung Cancer. Annu. Rev. Med. 2020, 71, 117–136. 10.1146/annurev-med-051718-013524. 31986082

[ref30] Rosell R.; Carcereny E.; Gervais R.; Vergnenegre A.; Massuti B.; Felip E.; Palmero R.; Garcia-Gomez R.; Pallares C.; Sanchez J. M.; et al. Erlotinib versus standard chemotherapy as first-line treatment for European patients with advanced EGFR mutation-positive non-small-cell lung cancer (EURTAC): a multicentre, open-label, randomised phase 3 trial. Lancet Oncol. 2012, 13, 239–246. 10.1016/S1470-2045(11)70393-X. 22285168

[ref31] Lim S. M.; Syn N. L.; Cho B. C.; Soo R. A. Acquired resistance to EGFR targeted therapy in non-small cell lung cancer: Mechanisms and therapeutic strategies. Cancer Treat. Rev. 2018, 65, 1–10. 10.1016/j.ctrv.2018.02.006. 29477930

[ref32] Chen Y.-J.; Roumeliotis T. I.; Chang Y.-H.; Chen C.-T.; Han C.-L.; Lin M.-H.; Chen H.-W.; Chang G.-C.; Chang Y.-L.; Wu C.-T.; et al. Proteogenomics of Non-smoking Lung Cancer in East Asia Delineates Molecular Signatures of Pathogenesis and Progression. Cell 2020, 182, 226–244. 10.1016/j.cell.2020.06.012. 32649875

[ref33] Lee J. E.; Park H. S.; Lee D.; Yoo G.; Kim T.; Jeon H.; Yeo M.-K.; Lee C.-S.; Moon J. Y.; Jung S. S.; et al. Hippo pathway effector YAP inhibition restores the sensitivity of EGFR-TKI in lung adenocarcinoma having primary or acquired EGFR-TKI resistance. Biochem. Biophys. Res. Commun. 2016, 474, 154–160. 10.1016/j.bbrc.2016.04.089. 27105908

[ref34] Hsu P.-C.; You B.; Yang Y.-L.; Zhang W.-Q.; Wang Y.-C.; Xu Z.; Dai Y.; Liu S.; Yang C.-T.; Li H.; et al. YAP promotes erlotinib resistance in human non-small cell lung cancer cells. Oncotarget 2016, 7, 51922–51933. 10.18632/oncotarget.10458. 27409162 PMC5239524

[ref35] Zhang X.; Maity T.; Kashyap M. K.; Bansal M.; Venugopalan A.; Singh S.; Awasthi S.; Marimuthu A.; Charles Jacob H. K.; Belkina N.; et al. Quantitative Tyrosine Phosphoproteomics of Epidermal Growth Factor Receptor (EGFR) Tyrosine Kinase Inhibitor-treated Lung Adenocarcinoma Cells Reveals Potential Novel Biomarkers of Therapeutic Response. Mol. Cell. Proteomics 2017, 16, 891–910. 10.1074/mcp.M117.067439. 28331001 PMC5417828

[ref36] Lan T.; Li Y.; Wang Y.; Wang Z.-C.; Mu C.-Y.; Tao A.-B.; Gong J.-L.; Zhou Y.; Xu H.; Li S.-B.; et al. Increased endogenous PKG I activity attenuates EGF-induced proliferation and migration of epithelial ovarian cancer via the MAPK/ERK pathway. Cell Death Dis. 2023, 14, 39 10.1038/s41419-023-05580-y. 36653376 PMC9849337

[ref37] Engelman J. A.; Zejnullahu K.; Mitsudomi T.; Song Y.; Hyland C.; Park J. O.; Lindeman N.; Gale C.-M.; Zhao X.; Christensen J.; et al. MET amplification leads to gefitinib resistance in lung cancer by activating ERBB3 signaling. Science 2007, 316, 1039–1043. 10.1126/science.1141478. 17463250

[ref38] Quesnelle K. M.; Boehm A. L.; Grandis J. R. STAT-mediated EGFR signaling in cancer. J. Cell. Biochem. 2007, 102, 311–319. 10.1002/jcb.21475. 17661350

[ref39] Zheng Y.; Xia Y.; Hawke D.; Halle M.; Tremblay M. L.; Gao X.; Zhou X. Z.; Aldape K.; Cobb M. H.; Xie K.; et al. FAK phosphorylation by ERK primes ras-induced tyrosine dephosphorylation of FAK mediated by PIN1 and PTP-PEST. Mol. Cell 2009, 35, 11–25. 10.1016/j.molcel.2009.06.013. 19595712 PMC2715139

[ref40] Dubois F.; Bergot E.; Zalcman G.; Levallet G. RASSF1A, puppeteer of cellular homeostasis, fights tumorigenesis, and metastasis-an updated review. Cell Death Dis. 2019, 10, 928 10.1038/s41419-019-2169-x. 31804463 PMC6895193

[ref41] Bae S. J.; Ni L.; Osinski A.; Tomchick D. R.; Brautigam C. A.; Luo X. SAV1 promotes Hippo kinase activation through antagonizing the PP2A phosphatase STRIPAK. eLife 2017, 6, e30278 10.7554/eLife.30278. 29063833 PMC5663475

[ref42] Sinclear C. K.; Maruyama J.; Nagashima S.; Arimoto-Matsuzaki K.; Kuleape J. A.; Iwasa H.; Nishina H.; Hata Y. Protein kinase Cα activation switches YAP1 from TEAD-mediated signaling to p73-mediated signaling. Cancer Sci. 2022, 113, 1305–1320. 10.1111/cas.15285. 35102644 PMC8990296

[ref43] Ando T.; Arang N.; Wang Z.; Costea D. E.; Feng X.; Goto Y.; Izumi H.; Gilardi M.; Ando K.; Gutkind J. S. EGFR Regulates the Hippo pathway by promoting the tyrosine phosphorylation of MOB1. Commun. Biol. 2021, 4, 1237 10.1038/s42003-021-02744-4. 34725466 PMC8560880

[ref44] Lee T.-F.; Tseng Y.-C.; Nguyen P. A.; Li Y.-C.; Ho C.-C.; Wu C.-W. Enhanced YAP expression leads to EGFR TKI resistance in lung adenocarcinomas. Sci. Rep. 2018, 8, 271 10.1038/s41598-017-18527-z. 29321482 PMC5762715

[ref45] Shimizu Y.; Okada K.; Adachi J.; Abe Y.; Narumi R.; Uchibori K.; Yanagitani N.; Koike S.; Takagi S.; Nishio M.; Fujita N.; Katayama R. GSK3 inhibition circumvents and overcomes acquired lorlatinib resistance in ALK-rearranged non-small-cell lung cancer. NPJ. Precis. Oncol. 2022, 6, 16 10.1038/s41698-022-00260-0. 35301419 PMC8931094

[ref46] Pozo K.; Bibb J. A. The emerging role of cdk5 in cancer. Trends Cancer 2016, 2, 606–618. 10.1016/j.trecan.2016.09.001. 27917404 PMC5132345

[ref47] Okuda S.; Watanabe Y.; Moriya Y.; Kawano S.; Yamamoto T.; Matsumoto M.; Takami T.; Kobayashi D.; Araki N.; Yoshizawa A. C.; et al. jPOSTrepo: an international standard data repository for proteomes. Nucleic Acids Res. 2017, 45, D1107–D1111. 10.1093/nar/gkw1080. 27899654 PMC5210561

