# Supplementary material for: A Rapid One-Pot Workflow for Sensitive Microscale Phosphoproteomics
Source: J Proteome Res. 2024 Jul 22;23(8):3294–309. doi: 10.1021/acs.jproteome.3c00862 (PMC11301667; doi:10.1021/acs.jproteome.3c00862)
Supplement: Supplementary file 1 — pr3c00862_si_001.pdf [file pr3c00862_si_001.pdf]

Supporting Information

A Rapid One-Pot Workflow for Sensitive Microscale Phosphoproteomics

Gul Muneer[1,2,3], Ciao-Syuan Chen[1], Tzu-Tsung Lee[1], Bo-Yu Chen[1], Yu-Ju Chen[1,3,5]*

[1]Institute of Chemistry, Academia Sinica, Taipei 11529, Taiwan

[2]Institute of Biochemical Sciences, National Taiwan University, Taipei 10617, Taiwan

[3]Chemical Biology and Molecular Biophysics Program, Taiwan International Graduate Program, Academia Sinica, Taipei 11529, Taiwan

[4]Department of Chemistry, National Taiwan University, Taipei 10617, Taiwan

*Corresponding author: yujuchen@gate.sinica.edu.tw

Table of Contents

Supplementary Figure 1. (A) Comparison of total protein yields using different lysis buffer strategies for direct protein extraction from 5×10^6 PC9 cells. S4

Supplementary Figure 2. Optimization of tryptic digestion time for low input amount.. .... S5

Supplementary Figure 3. Extracted ion chromatogram and mass spectrum of DDM in (A) uncoated, (B) partially coated, (C) fully coated experimental conditions. S6

Supplementary Figure 4. (A) Overlap between phosphopeptides identified by samples prepared in uncoated and fully-coated DDM-tubes. S7

Supplementary Figure 5. Comparison of identification and quantitation performance of phosphoproteomics of low input amount (10 µg and 5 µg) by DIA and DDA methods. S8

Supplementary Figure 6. Overlapped phosphopeptides identified from 5 µg - 0.5 µg input amount using dirDIA, large-library and small-library. S9

Supplementary Figure 7. (A-D) Comparison of distribution of q-values (FDR) of detected precursors and distribution of localization probabilities between dirDIA and lib-based DIA from 0.5 µg and 5 µg sample input.. .... S10

Supplementary Figure 8. Distribution of FDR based on localization probability score from 0.5 µg (left) and 5 µg (right) sample input by (A-B) dirDIA and (C-D) libDIA. S11

Supplementary Figure 9. The MS1 mass accuracy tolerance (left) and distribution of mass errors (right) of 0.5 µg sample input from (A) dirDIA, (B) large libDIA, and (C) small libDIA. S12

Supplementary Figure 10. Summary of FDR and localization probabilities by target and decoy search using dirDIA. S13

Supplementary Figure 11. Summary of FDR and localization probabilities by target and decoy search using libDIA. S14

Supplementary Figure 12. Summary of monoisotopic distribution, fragment ion spectra and extracted ion chromatogram (XIC) of phosphopeptides that are uniquely detected from small libDIA. S15

Supplementary Figure 13. Overlapped phosphopeptides identified from 2500 cells - 50000 cells input amount using dirDIA, large-library and small-library. S16

Following supporting information files (XLSX) are provided as separate files.

Table S1. Summary of identification results from different protocols **(XLSX)**.

Table S2. Summary of identification results from conventional, 4 hours, 2 hours and 1 **(XLSX)**.

Table S3. Summary of identification results DDM coating on peptide recovery **(XLSX)**.

Table S4. Summary of identification results from low input amount by DIA and DDA **(XLSX)**.

Table S5. Summary of identification results from direct DIA **(XLSX).**

Table S6. Summary of identification results from library DIA **(XLSX).**

Table S7. Summary of identification results from direct DIA **(XLSX).**

Table S8. Summary of identification results from library DIA **(XLSX).**

Table S9. Summary of identification results from PC9, CL68, H1975 and H3255 cells **(XLSX).**

Table S10. Summary of KEGG pathway analysis of phosphoproteins from 1000 to 10 cells **(XLSX).**

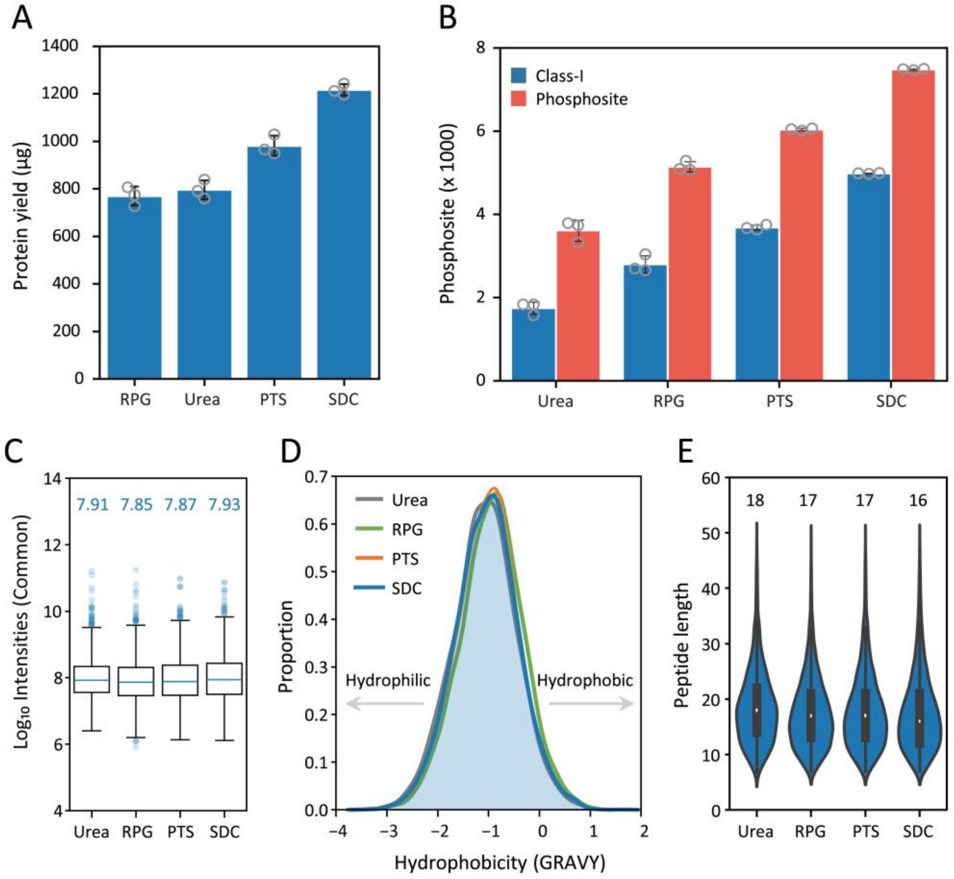

Supplementary Figure 1. (A) Comparison of total protein yields using different lysis buffer strategies for direct protein extraction from 5×10^6 PC9 cells. **(B)** Number of phosphosites and class-1 (localization probability ≥ 0.75) identified by each sample preparation workflow. **(C)** Distribution of $\log_{10}$-transformed intensities of commonly quantified phosphopeptides. **(D)** Distribution of peptide hydrophobicity (GRAVY) for quantified phosphopeptides. **(E)** Violin plot showing distribution of peptide length by different sample preparation workflows.

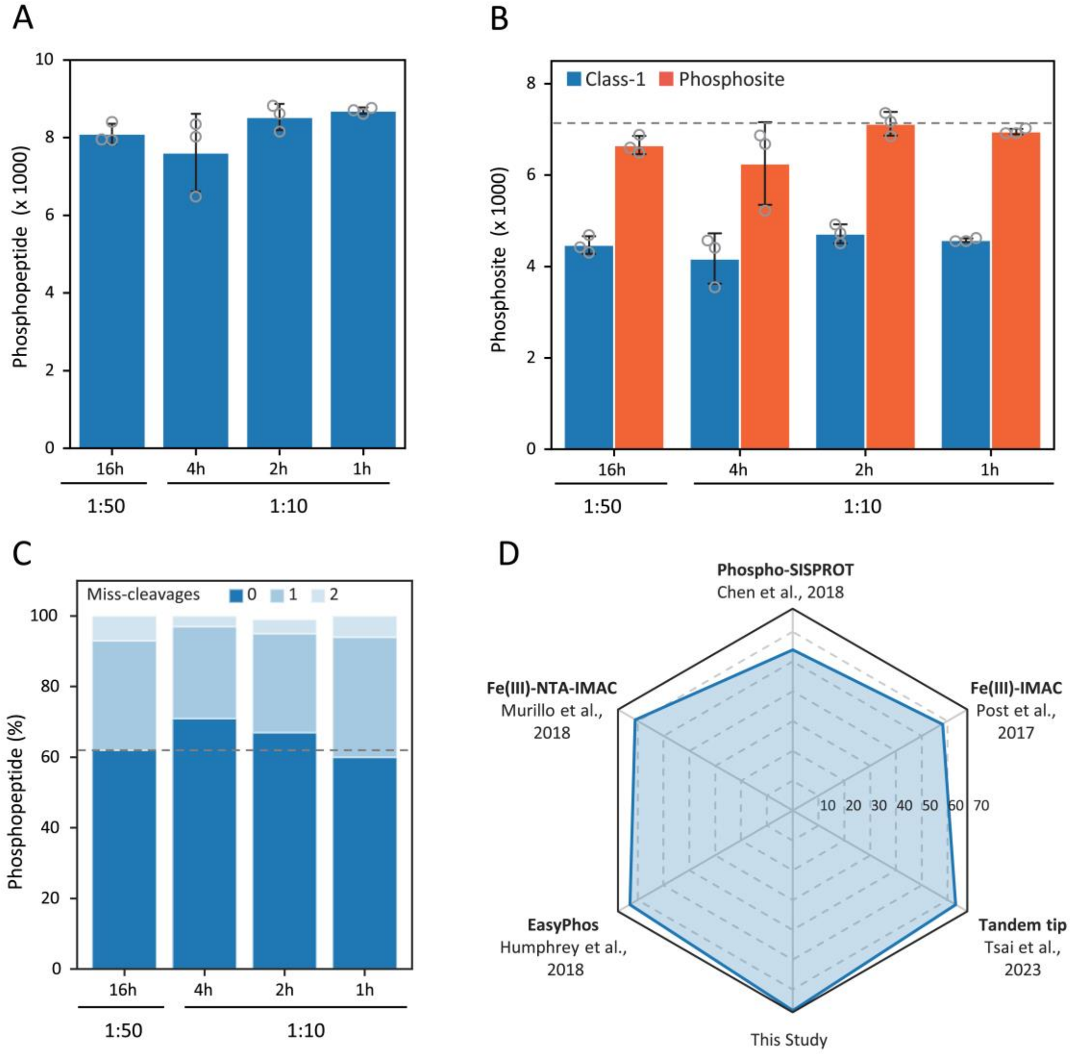

Supplementary Figure 2. Optimization of tryptic digestion time for low input amount. (A-B) Summary of phosphopeptides and phosphosites detected after conventional (16 hours digestion), 4 hours, 2 hours and 1 hour digestion duration. **(C)** Proportion of missed cleavages after tryptic digestion at different digestion times. **(D)** Fraction of phosphopeptides containing 0 missed cleavages in this workflow compared to five others recently published phosphoproteome studies employing conventional digestion time.

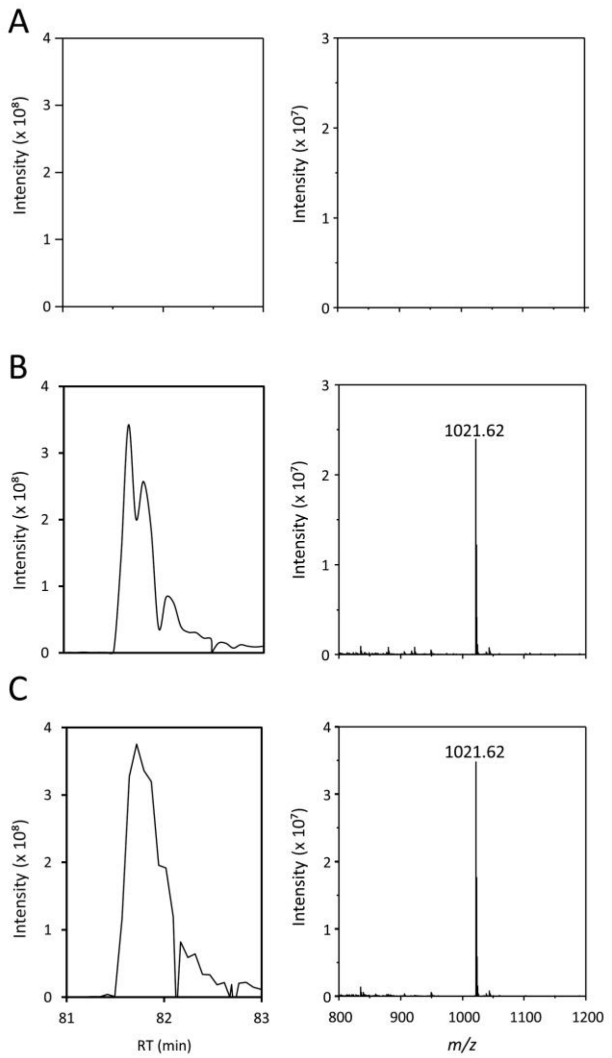

Supplementary Figure 3. Extracted ion chromatogram and mass spectrum of DDM in **(A)** uncoated, **(B)** partially coated, **(C)** fully coated experimental conditions.

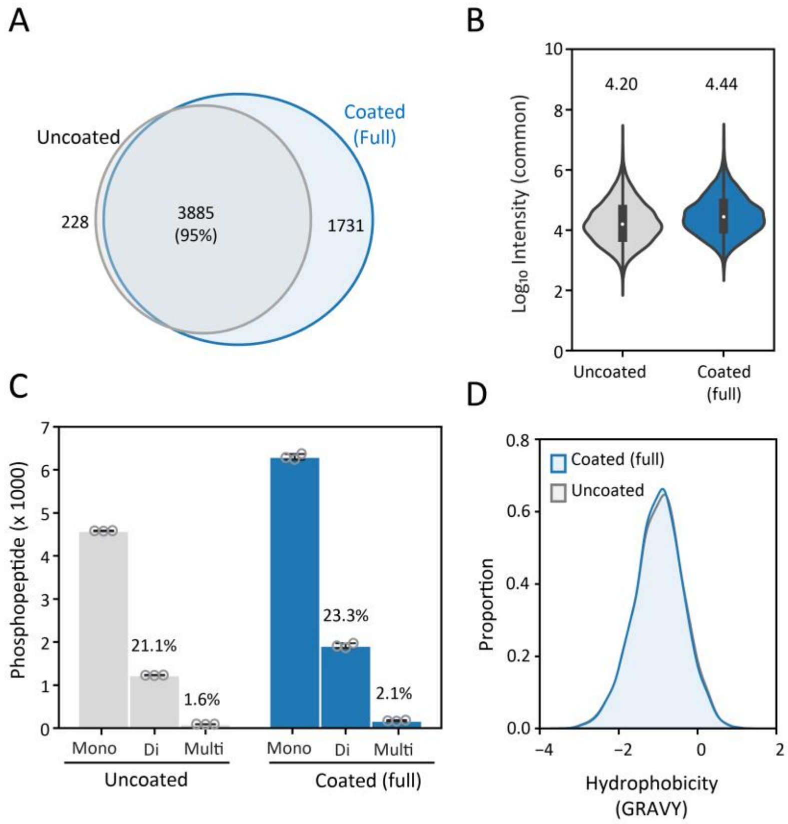

Supplementary Figure 4. (A) Overlap between phosphopeptides identified by samples prepared in uncoated and fully-coated DDM-tubes. **(B)** Violin plot showing the distributions of quantified phosphopeptides by each approach. **(C)** Summary of singly, di- and multiply phosphorylated peptides identified in uncoated and coated workflows. **(D)** Distribution of peptide hydrophobicity (GRAVY) for quantified phosphopeptides detected in samples prepared in uncoated and coated tubes.

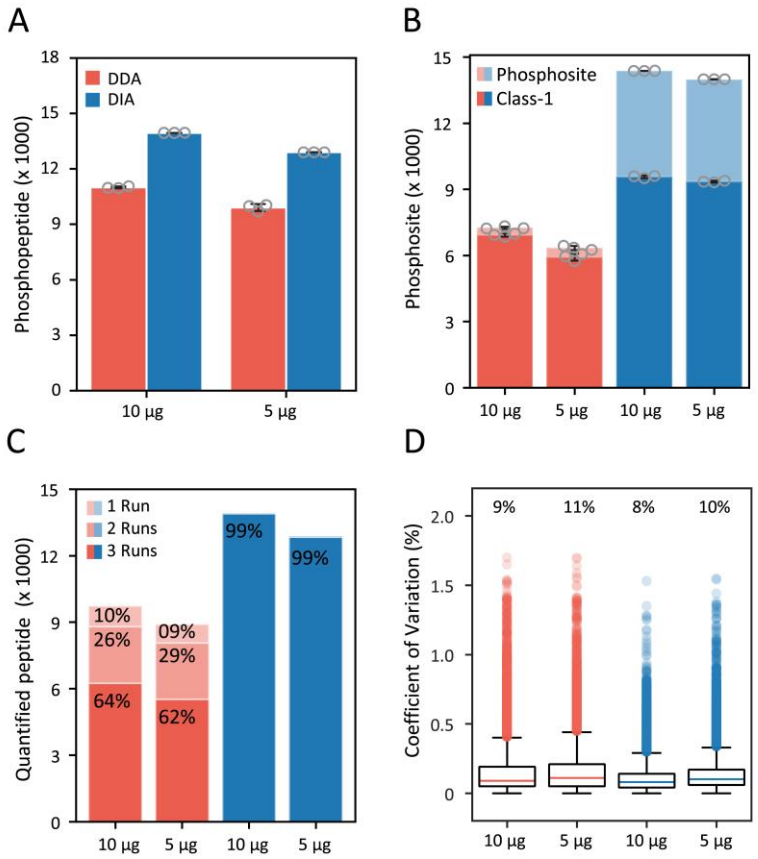

Supplementary Figure 5. Comparison of identification and quantitation performance of phosphoproteomics of low input amount (10 µg and 5 µg) by DIA and DDA methods. (A-B) Comparison of Phosphopeptides and phosphosites identified by triplicate analysis using DDA and DIA modes. **(C)** Evaluation of missing values (%) of phosphopeptides identified and quantified in each run by DDA and DIA method. **(D)** Distribution of coefficient of variation (CV%) for quantified phosphopeptides by both methods.

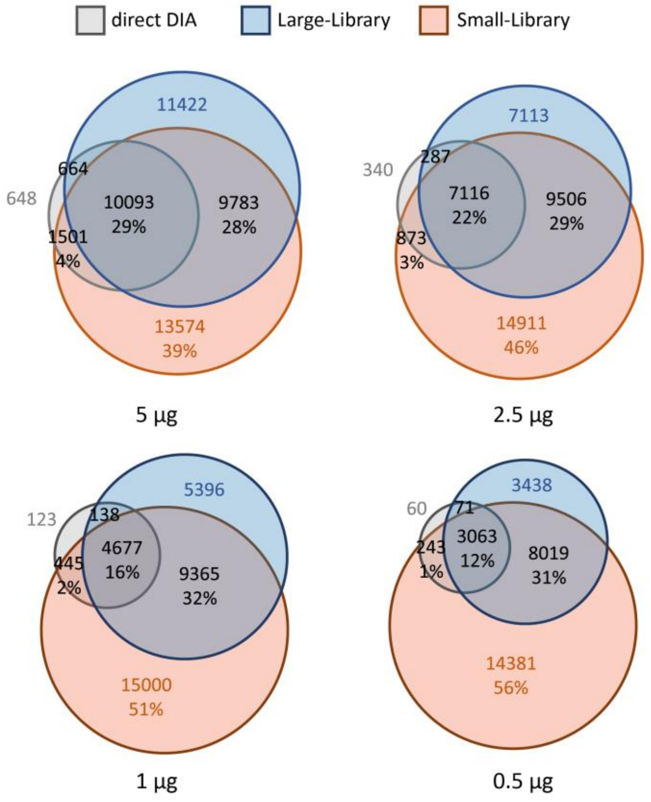

Supplementary Figure 6. Overlapped phosphopeptides identified from 5 μg - 0.5 μg input amount using dirDIA, large-library and small-library.

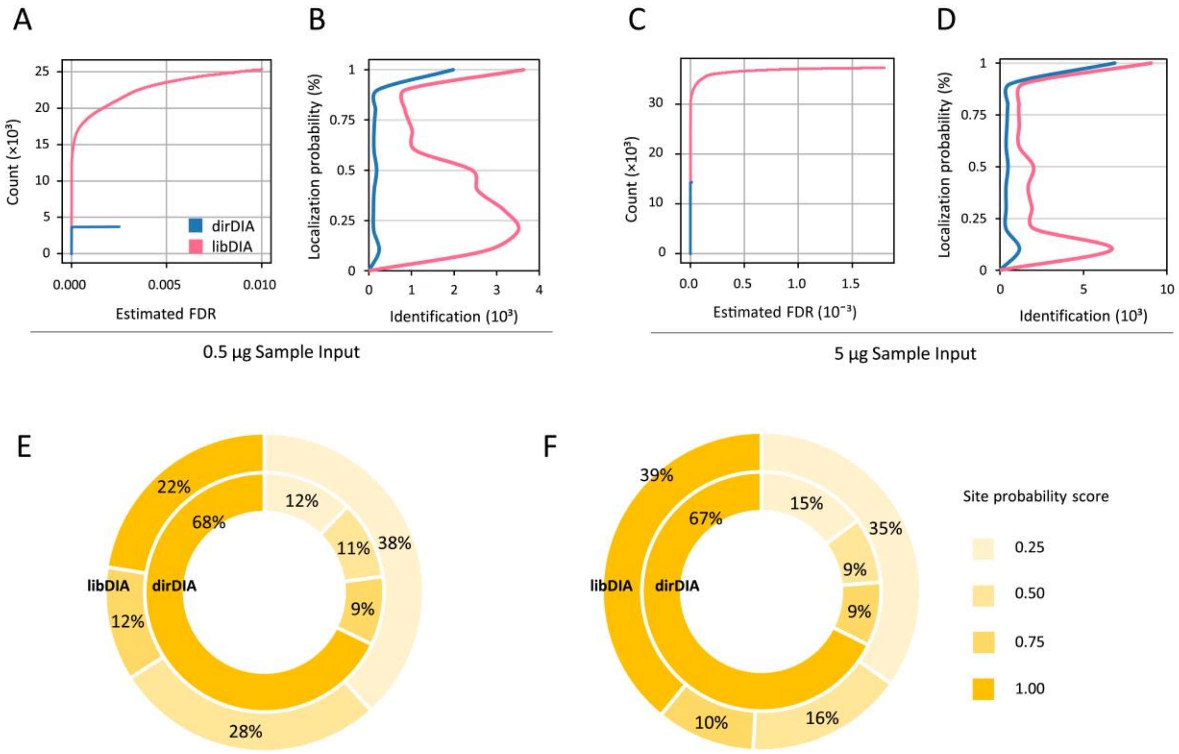

Supplementary Figure 7. (A-D) Comparison of distribution of q-values (FDR) of detected precursors and distribution of localization probabilities between dirDIA and lib-based DIA from 0.5 µg and 5 µg sample inputs. **(E-F)** Percentage of localization probabilities of phosphosites detected in dirDIA and libDIA from 0.5 µg and 5 µg sample inputs.

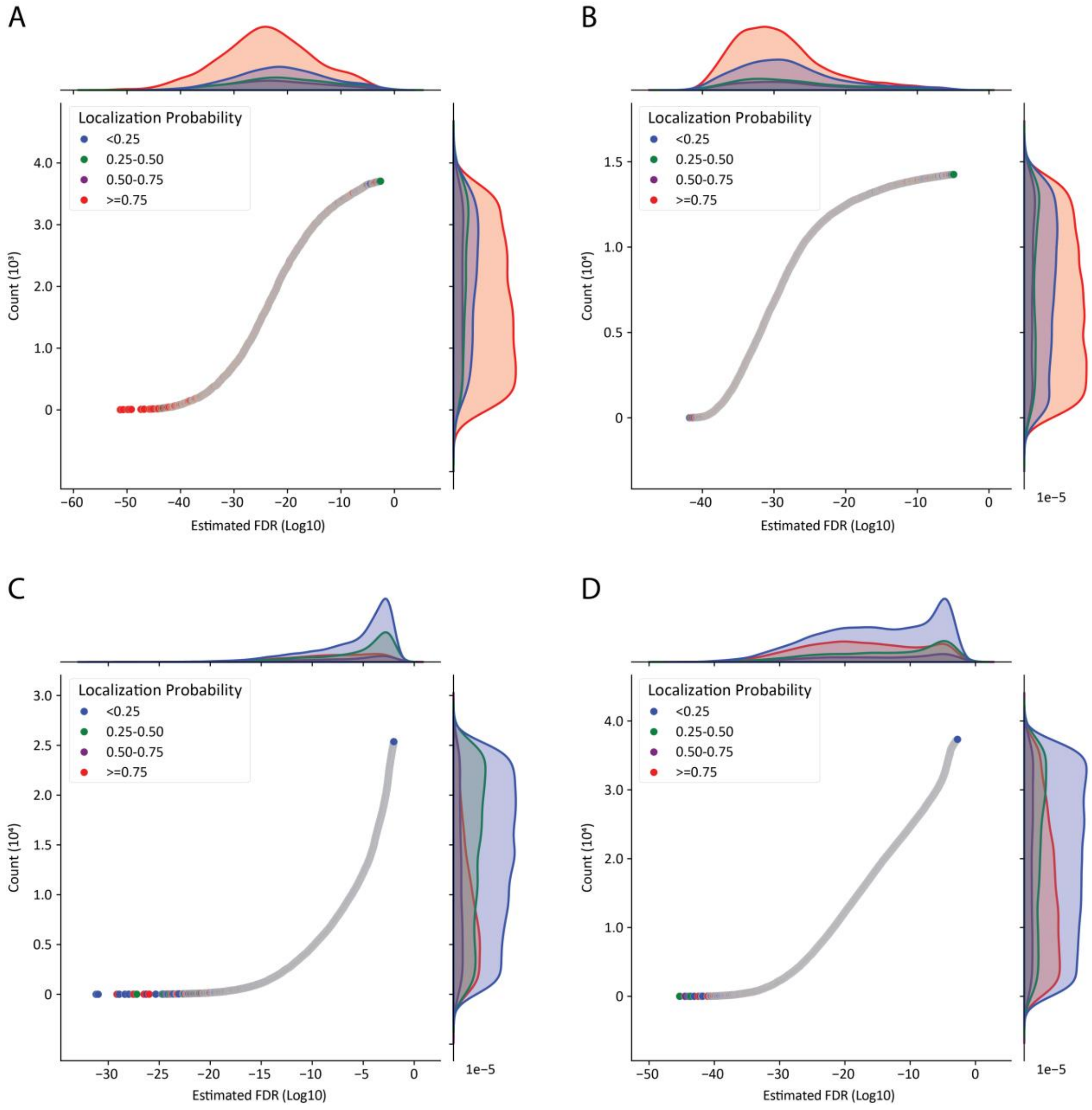

Supplementary Figure 8. Distribution of FDR based on localization probability score from 0.5 µg (left) and 5 µg (right) sample input by **(A-B)** dirDIA and **(C-D)** libDIA.

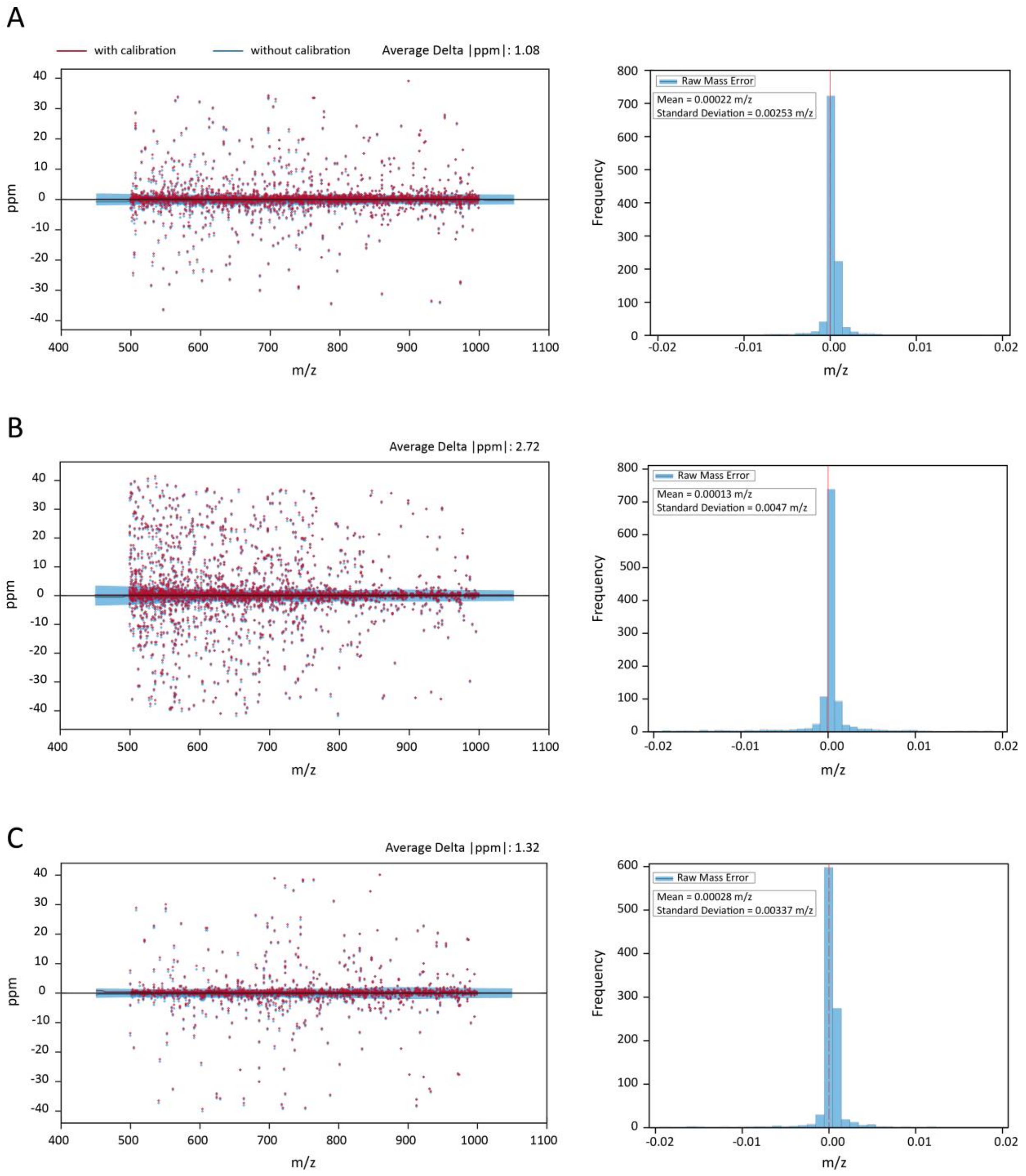

Supplementary Figure 9. The MS1 mass accuracy tolerance (left) and distribution of mass errors (right) of 0.5 µg sample input from **(A)** dirDIA, **(B)** large libDIA, and **(C)** small libDIA.

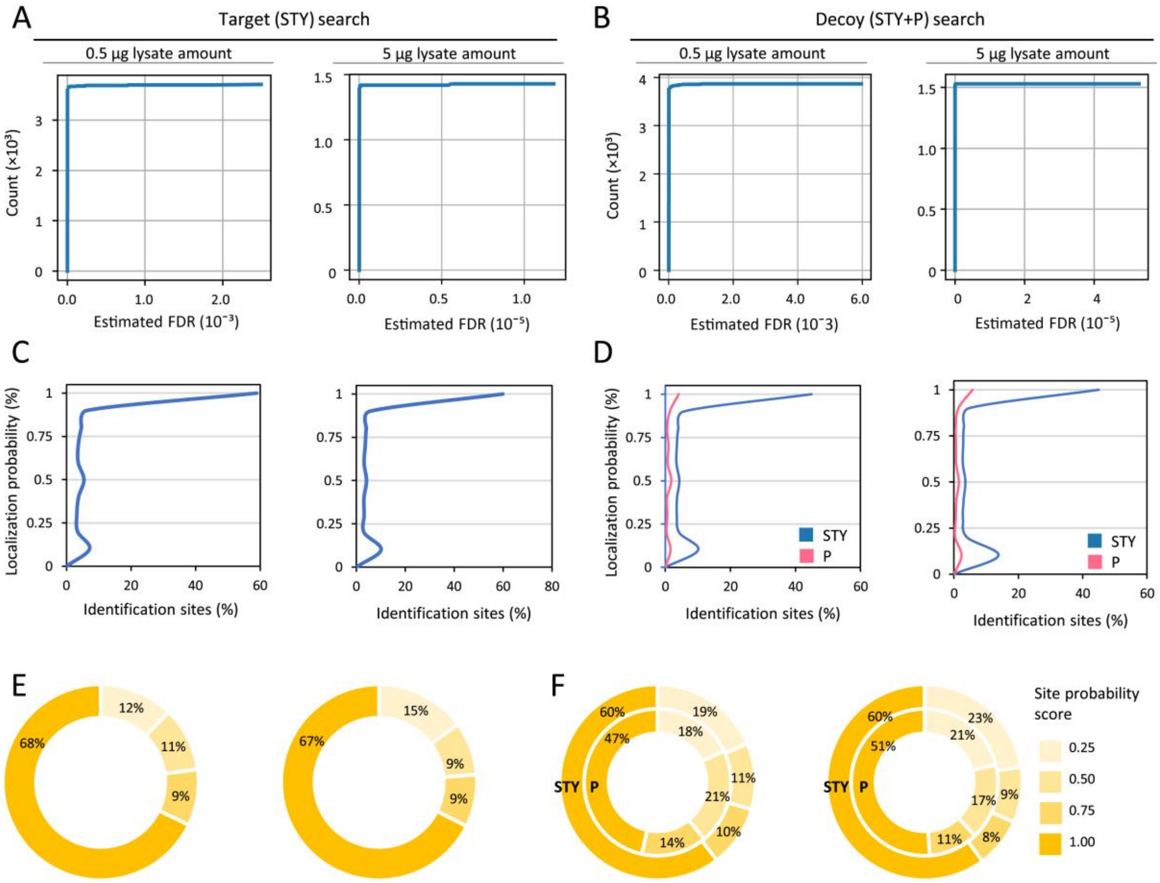

Supplementary Figure 10. Summary of FDR and localization probabilities by target and decoy search using dirDIA. (A-B) Distribution of q-values (FDR) of detected precursors by target and decoy search using 0.5 µg and 5 µg sample inputs. **(C)** Distribution of localization probabilities by target search. **(D)** Comparison of distribution of localization probabilities of S, T, Y and P residue by decoy search. **(E)** Percentage of localization probabilities of phosphosites detected by target search. **(F)** Comparison of percentage of localization probabilities of phosphosites detected by decoy search.

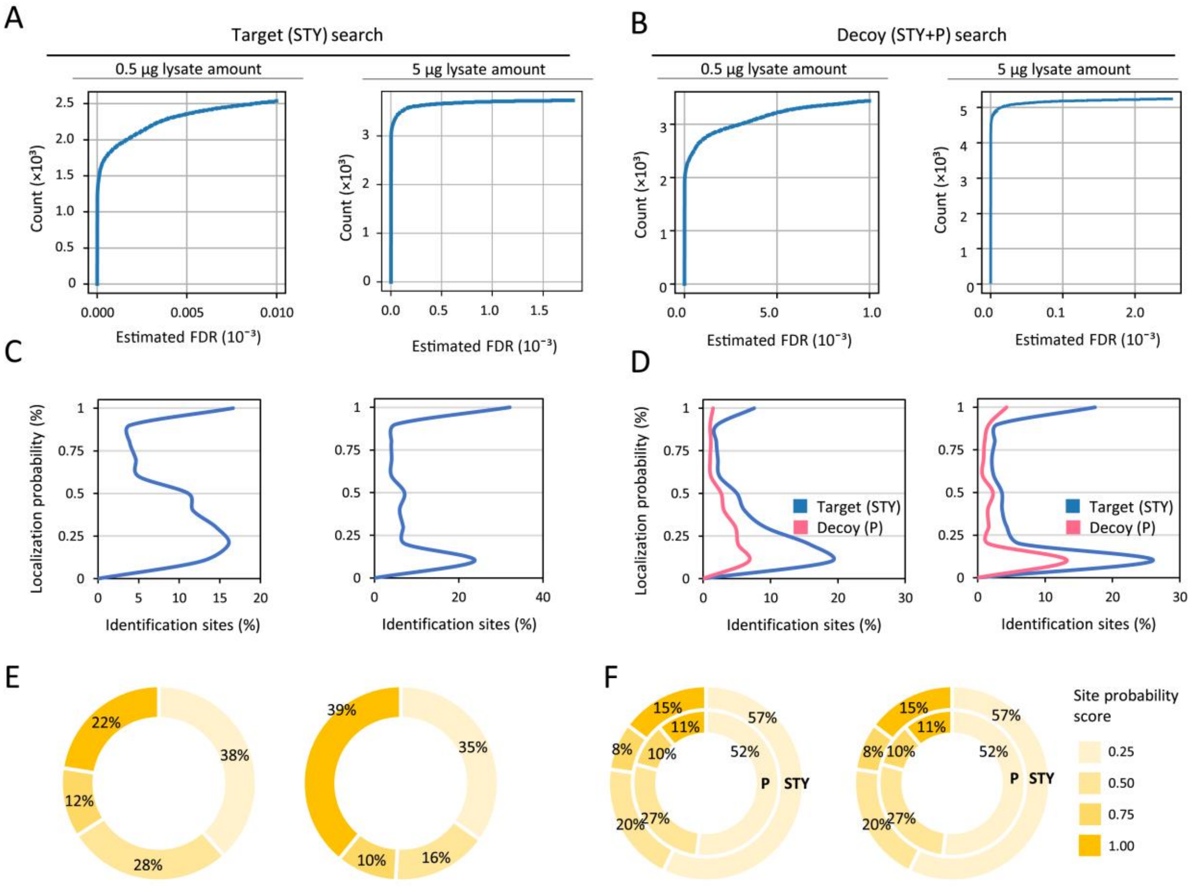

Supplementary Figure 11. Summary of FDR and localization probabilities by target and decoy search using libDIA. (A-B) Distribution of q-values (FDR) of detected precursors by target and decoy search using 0.5 µg and 5 µg sample inputs. **(C)** Distribution of localization probabilities by target search. **(D)** Comparison of distribution of localization probabilities of S, T, Y and P residue by decoy search. **(E)** Percentage of localization probabilities of phosphosites detected by target search. **(F)** Comparison of percentage of localization probabilities of phosphosites detected by decoy search.

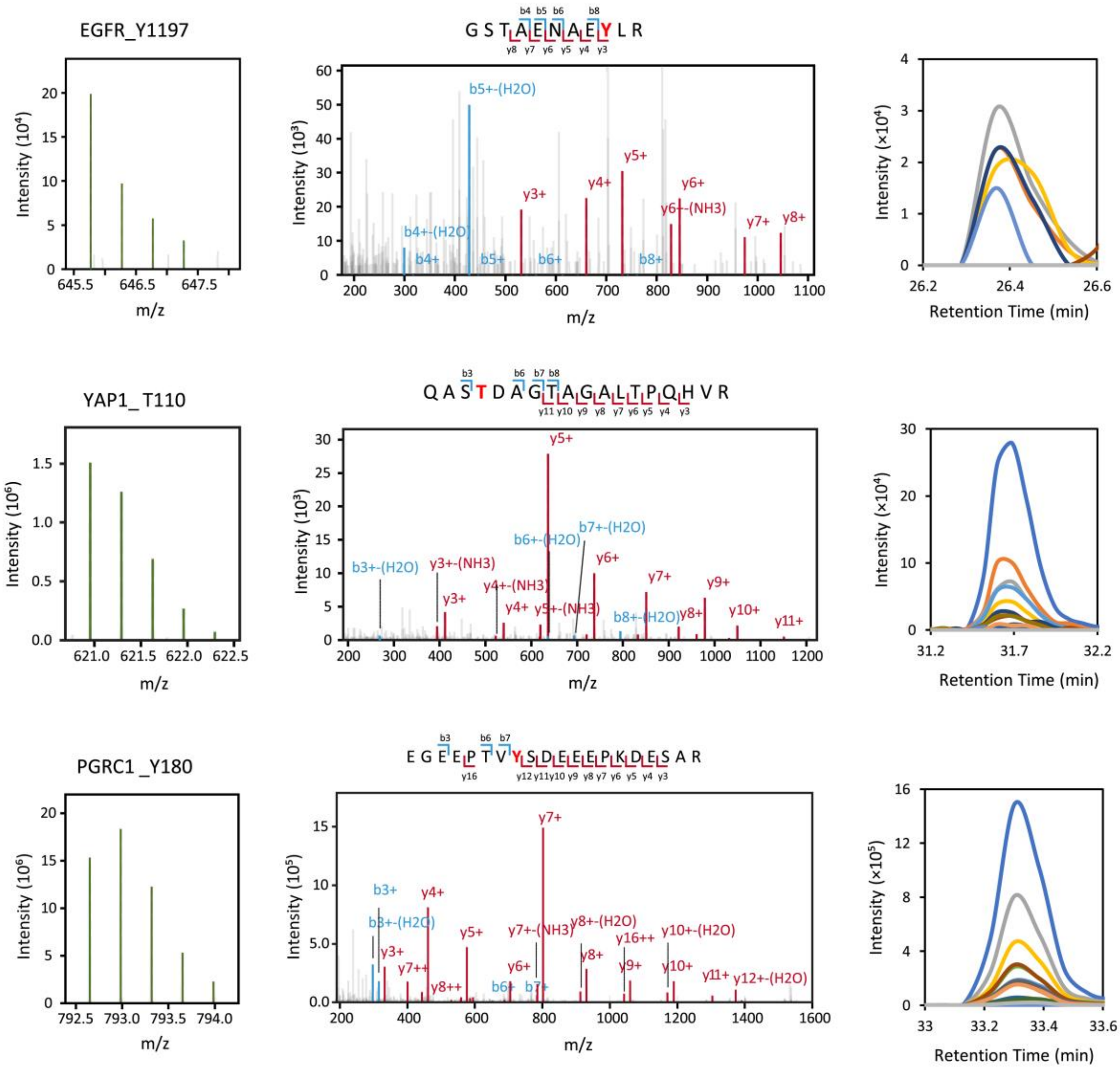

Supplementary Figure 12. Summary of monoisotopic distribution, fragment ion spectra and extracted ion chromatogram (XIC) of phosphopeptides that are uniquely detected from small libDIA.

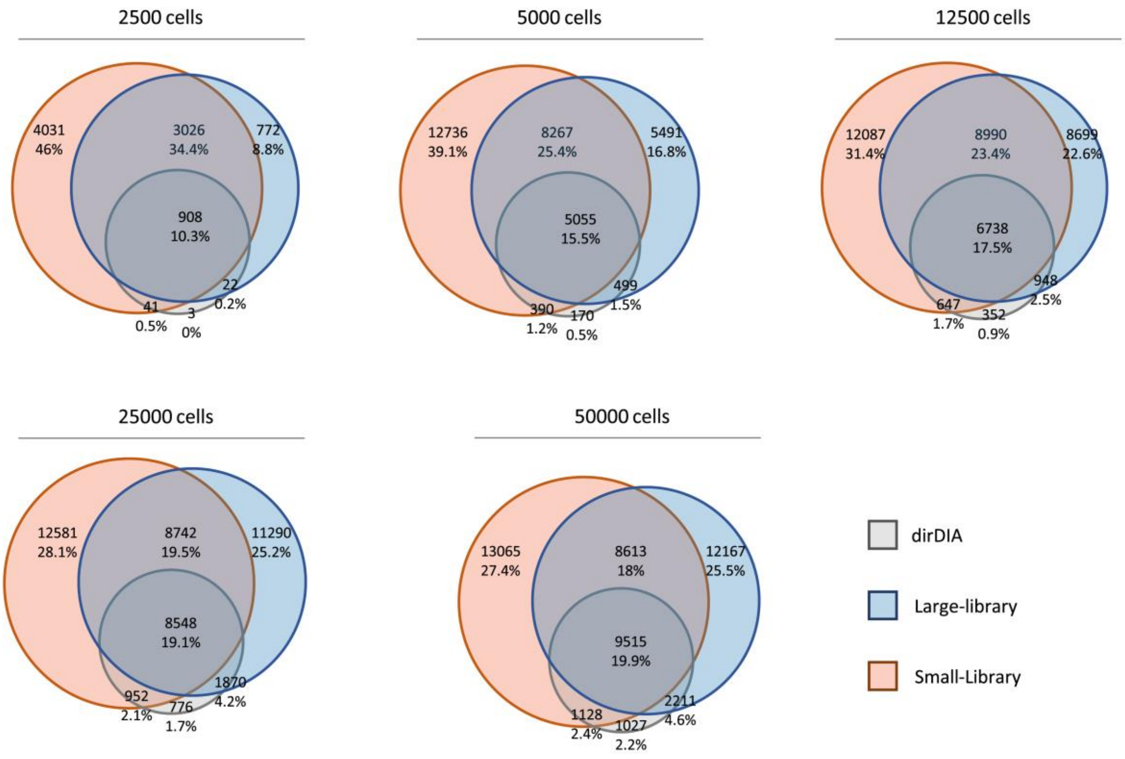

Supplementary Figure 13. Overlapped phosphopeptides identified from 2,500 cells – 50,000 cells input amount using dirDIA, large-library and small-library.
